# miRNAProtPred: computational prediction of human miRNA binding based on seed complementarity and thermodynamic stability

**DOI:** 10.3389/fgene.2026.1817432

**Published:** 2026-06-24

**Authors:** Somenath Dutta, Manisha Pritam, Sudipta Sardar, Nitimoy Mondal, Sun Gu Lee

**Affiliations:** 1 Department of Chemical and Biomolecular Engineering, Pusan National University, Busan, Republic of Korea; 2 Laboratory of Malaria Immunology and Vaccinology, NIAID, NIH, Bethesda, MD, United States; 3 CSIR-Indian Institute of Integrative Medicine, Jammu and Kashmir, India

**Keywords:** Antiviral miRNA, HIV-1, human miRNA, minimum free energy, miRNA-based therapy, SARS-CoV-2

## Abstract

**Introduction:**

Computational prediction of microRNA-target interactions is essential for understanding post-transcriptional regulation, yet existing tools often require manual data curation, lack comprehensive miRNA databases, or provide limited guidance for experimental prioritization.

**Methods:**

We developed miRNAProtPred, a Python package that consolidates established seed complementarity matching and ViennaRNA-based thermodynamic analysis into a streamlined workflow for predicting human miRNA binding sites on diverse target sequences. The tool integrates 2,656 curated human miRNAs from miRDB and miRBase, accepts diverse input formats (DNA, RNA, or protein sequences), and classifies predictions through a multi-criteria confidence framework incorporating seed complementarity, thermodynamic stability (minimum free energy, MFE), flanking AU content, motif identity, and match type. miRNAProtPred supports two user-selectable search modes: a default strict mode requiring exact Watson-Crick seed complementarity, and a relaxed mode that extends sensitivity to G:U wobble-supported interactions through a hierarchical exact-first fallback strategy. The tool was evaluated using experimentally validated antiviral miRNAs from SARS-CoV-2 and HIV-1, and independently benchmarked on the miRAW dataset (62,215 miRNA-target pairs).

**Results:**

For SARS-CoV-2, miRNAProtPred successfully identified all 16 experimentally supported inhibitory miRNAs compiled from multiple independent studies (100% recovery in strict mode), with 12 of 16 (75%) classified as high confidence (MFE ≤ −12 kcal/mol). For HIV-1, 11 of 13 (84.6%) validated miRNAs were identified through canonical seed matching, with 7 of 11 (63.6%) classified as high confidence; the remaining two miRNAs (hsa-miR-92a-3p and hsa-miR-382-5p) were recovered through the wobble-permissive relaxed mode, achieving complete recovery across both viral systems. Large-scale evaluation on the miRAW benchmark dataset confirmed the precision-oriented performance profile, with strict mode achieving 98.66% precision, 73.97% recall, an F1-score of 0.846, and a Matthews correlation coefficient of 0.748. Validated miRNAs showed thermodynamic enrichment compared to genome-wide predictions (SARS-CoV-2: −11.87 vs. −9.695 kcal/mol; HIV-1: −12.13 vs. −11.215 kcal/mol), supporting MFE-based prioritization.

**Discussion:**

miRNAProtPred provides a streamlined, pip-installable tool for predicting human miRNA binding sites on diverse target sequences, facilitating candidate prioritization for experimental validation. The package is freely available at https://github.com/somenath-combio/mirnaprotpred.

## 1 Introduction

MicroRNAs (miRNAs) are endogenous ∼22-nucleotide non-coding RNAs that regulate gene expression through post-transcriptional mechanisms. The discovery of miRNA as a key regulator in post-transcriptional gene expression, first demonstrated by Victor Ambros and Gary Ruvkun in *Caenorhabditis elegans*, established the foundation for understanding miRNA’s role in diverse biological processes including development, immunity, and disease (Lee et al., 1993; Almeida et al., 2011). When a miRNA binds to messenger RNA (mRNA), it induces either translational repression or mRNA degradation, primarily through Watson-Crick base pairing between the miRNA seed region (nucleotides 2–8 at the 5′ end) and complementary target sites (Valinezhad Orang et al., 2014). miRNA-mRNA interactions regulate fundamental cellular processes in both physiological and pathological contexts, including cancer progression, metabolic homeostasis, neurological disorders, and host-pathogen interactions (Lei et al., 2022; Diener et al., 2024). In the context of infectious diseases, specific human miRNAs can bind directly to pathogen-derived RNA, including the genomic RNA of positive-sense single-stranded RNA (+ssRNA) viruses, thereby inhibiting pathogen gene expression and replication (Lei et al., 2022; Xu et al., 2022). In addition, human miRNAs can contribute to antiviral defense indirectly by regulating the expression of host factors required for viral entry, replication, or immune evasion, underscoring the dual role of miRNAs in direct viral suppression and host-pathway regulation (Trobaugh and Klimstra, 2017; Dutta et al., 2026). Representative examples include hsa-miR-29a targeting the Nef gene of Human immunodeficiency virus-1 (HIV-1), hsa-miR-92a-3p targeting the PfEMP1 gene of Plasmodium falciparum, and mosquito miR-252 from *Aedes albopictus* targeting the envelope gene of dengue virus serotype 2 (DENV-2), demonstrating that miRNA-based regulation extends beyond endogenous gene control to encompass diverse molecular targets (Yan et al., 2014; Frattari et al., 2017; Prabhu et al., 2023).

To identify therapeutically relevant miRNA-target interactions, computational prediction tools have become indispensable, particularly given the impracticality of experimentally screening thousands of miRNAs against target transcriptomes (Riolo et al., 2020). The clinical relevance of computational miRNA target prediction has been validated through translational successes such as Miravirsen, an anti-miR-122 therapeutic for hepatitis C virus that advanced to Phase II clinical trials following computational identification of miR-122-target interactions (Ottosen et al., 2015; van der Ree et al., 2016). Computational approaches for miRNA target prediction have evolved along several methodological trajectories (Riolo et al., 2020). Seed-based methods identify canonical complementarity between miRNA seed regions and target sites, leveraging the well-established biological mechanism of miRNA recognition (Wang, 2014). This approach can be very generally applied to any target, but its sensitivity is relatively poor. Energy-based approach can be combined to overcome the limitation by evaluating thermodynamic stability of miRNA-mRNA duplexes using minimum free energy (MFE) calculations (Hammell, 2010). Machine learning-based methods integrate sequence features, structural accessibility, and evolutionary conservation to predict functional interactions, demonstrating success in identifying targets within human and model organism transcriptomes (Parveen et al., 2020; Ben Or and Veksler-Lublinsky, 2021). For instance, deep learning architectures employing convolutional neural networks have shown promise for capturing complex sequence patterns from large-scale validated datasets (Cheng et al., 2016; Wen et al., 2018). The sensitivity of machine-learning based approaches is relatively high, but the requirement of high-quality and experimentally validated training datasets limits their applications to the targets with limited data (Riolo et al., 2020).

Most previously published miRNA target prediction tools were specifically designed and optimized for identifying targets within human or model organism transcriptomes, with primary focus on 3′ untranslated regions (3′UTRs) and evolutionarily conserved sites across related species (Peterson et al., 2014). While platforms such as miRanda and RNAhybrid provide thermodynamic scoring capabilities, they require users to manually compile and format miRNA sequences before analysis (John et al., 2004; Kruger and Rehmsmeier, 2006). miRDB, through its MirTarget v4 algorithm, supports both canonical and non-canonical target prediction for human and a limited number of model organism transcriptomes (Chen and Wang, 2020); however, it does not accept user-supplied target sequences for arbitrary pathogens or cross-species analyses, limiting its application to viral or non-model-organism targets. Furthermore, no existing tool supports protein sequence input with automated conversion to corresponding nucleotide sequences, limiting accessibility for researchers working with protein annotation databases. Beyond these individual feature gaps, a critical practical barrier lies in the cumulative preprocessing burden imposed on users. For a typical miRNA target prediction analysis using miRanda or RNAhybrid, the researcher must independently obtain mature miRNA sequences from external databases (e.g., miRBase), compile them into correctly formatted FASTA files, prepare the target sequence in the required orientation and format, execute the prediction for each miRNA-target pair (or write custom batch-processing scripts), parse tool-specific output formats, and apply *post hoc* filtering thresholds to prioritize candidates. Each of these steps introduces potential for formatting errors, version mismatches between miRNA databases and prediction software, and laboratory-to-laboratory variation in filtering criteria, collectively undermining reproducibility (Riffo-Campos et al., 2016). The absence of a standardized, self-contained workflow that eliminates these manual steps represents a practical gap distinct from algorithmic innovation, yet one with direct consequences for the accessibility and reproducibility of miRNA target prediction in non-model-organism and cross-species contexts.

In this study, we present miRNAProtPred, a Python package designed to address this workflow-integration gap by consolidating two well-established methodological principles, canonical seed complementarity matching and ViennaRNA-based thermodynamic evaluation, into a single pip-installable, locally executable package that eliminates the manual preprocessing steps described above. The tool does not introduce new algorithmic machinery; rather, its contribution lies in three areas of practical integration that are not jointly available in any existing tool. First, miRNAProtPred bundles a curated database of 2,656 human miRNAs compiled from miRDB and miRBase, enabling genome-wide screening of all catalogued human miRNAs against any user-supplied target in a single command, without requiring the user to obtain, format, or maintain external miRNA sequence files. Second, the package accepts input in any of three formats (DNA, RNA, or protein), automatically detects the sequence type, and for protein inputs performs automated NCBI tblastn conversion to nucleotide sequence, a capability absent from all currently available miRNA target prediction tools and particularly relevant for researchers working with protein-centric annotation databases such as UniProt. Third, predictions are automatically classified into confidence tiers (High/Medium/Low) based on a multi-criteria framework incorporating thermodynamic stability, flanking AU content, motif identity, and match type, and exported with miRBase accession numbers in a structured CSV format, providing a standardized output that facilitates cross-study comparison and downstream prioritization without requiring users to define *ad hoc* filtering criteria.

We validated miRNAProtPred using SARS-CoV-2 and HIV-1 as model systems, comparing its predictions against experimentally verified antiviral miRNAs compiled from several independent studies, and independently evaluated its classification performance on a large-scale benchmark dataset of 62,215 experimentally validated miRNA-target interactions. The choice of a seed-based approach for these viral targets reflects a practical constraint: whereas databases such as miRTarBase contain thousands of validated human miRNA-human mRNA interactions suitable for training machine-learning classifiers in endogenous gene regulation contexts, comparable validated datasets for cross-species interactions, viral pathogens, and other non-canonical targets remain sparse (Cui et al., 2025). Until sufficiently large and diverse training data sets become available for these biological contexts, seed-based prediction complemented by thermodynamic evaluation remains a practical and broadly applicable approach whose underlying principles are supported by extensive experimental evidence (Lewis et al., 2005; Agarwal et al., 2015).

## 2 Materials and methods

### 2.1 Data retrieval and curation

Human miRNA data, including miRNA identifiers, names, mature sequences, and seed sequences (nucleotides 2-8 of the mature miRNA 5′ end), were retrieved from miRDB (https://mirdb.org/) and miRBase (https://mirbase.org/browse/results/?organism=hsa) (Kozomara et al., 2019). miRBase was used to obtain miRBase accession numbers for all human miRNAs. An automated Python-based pipeline was developed to systematically retrieve and integrate data from these databases, as neither platform provides a comprehensive downloadable dataset linking miRNA sequences, seed regions, and accession identifiers in a single file. The pipeline systematically queried miRDB with each human miRNA identifier from miRBase, extracted relevant information across multiple pages where necessary, and compiled the data into a structured format. Retrieved data underwent preprocessing to ensure quality and consistency. This included removal of whitespace, elimination of duplicate entries, and standardization of formatting conventions. The final curated dataset containing 2,656 human miRNAs was stored in a structured CSV format within the package for efficient pattern-matching operations during prediction analysis.

### 2.2 Validation dataset compilation

To validate the predictive accuracy of miRNAProtPred, we compiled reference datasets from two well-characterized viral pathogens: SARS-CoV-2 and HIV-1. The 3′UTR region of HIV-1 (nucleotides 8,631–9,085; 455 bp) was retrieved from NCBI database (https://www.ncbi.nlm.nih.gov/nuccore/) using Entry ID NC_001802.1. The 3′UTR region of SARS-CoV-2 isolate Wuhan-Hu-1 (nucleotides 29,675–29,903; 229 bp) was retrieved using Entry ID NC_045512.2 (Wu et al., 2020). Complete proteome sequences were obtained from the UniProt database (https://www.uniprot.org/proteomes): HIV-1 (UP000107373) and SARS-CoV-2 (UP000464024). Experimentally validated antiviral miRNAs were compiled from published studies identified through systematic PubMed database searches using the following keyword combinations: (“miRNA” OR “microRNA”) AND (“SARS-CoV-2” OR “COVID-19” OR “HIV-1”) AND (“inhibit” OR “suppress” OR “antiviral” OR “restrict”). For SARS-CoV-2, we included miRNAs with experimental evidence of direct antiviral activity through reporter assays, viral replication inhibition, or protein expression suppression (Barreda-Manso et al., 2021; Siniscalchi et al., 2021; Wang et al., 2021; Vaddadi et al., 2023). For HIV-1, we included miRNAs experimentally demonstrated to inhibit viral replication, reduce viral protein production, or suppress viral infectivity in CD4^+^ T cells or other relevant cell types (Huang et al., 2007; Ahluwalia et al., 2008; Nathans et al., 2009; Houzet et al., 2012; Swaminathan et al., 2012; Wang et al., 2015). For HIV-1 miRNAs reported in earlier studies without explicit arm designation (e.g., “miR-149,” “miR-92a,” “miR-150”), the canonical mature strand was assigned based on current miRBase annotation, consistent with the convention that unqualified miRNA names in the pre-2014 literature correspond to the dominant mature product catalogued as the guide strand (Kozomara et al., 2019). This compilation yielded 16 validated miRNAs for SARS-CoV-2 and 13 validated miRNAs for HIV-1, providing a reference dataset of functionally characterized host restriction factors for validating miRNAProtPred’s predictive accuracy.

### 2.3 Python package development

miRNAProtPred was developed as a pip-installable Python package to facilitate local installation and integration into bioinformatics workflows. The package was implemented in Python (version 3.9 or higher) and utilizes several core dependencies: pandas (version 2.3.3 or higher) for data management and tabular operations, Biopython (version 1.86 or higher) for sequence manipulation (Cock et al., 2009) and NCBI BLAST integration for automated protein to DNA conversion, and ViennaRNA (version 2.4.18 or higher) for minimum free energy calculation of secondary structure of RNA duplex formation (Lorenz et al., 2011). All dependencies are automatically installed during pip installation.

The package provides two command-line entry points. The primary SeqFinder module performs genome-wide miRNA target prediction by screening all 2,656 catalogued human miRNAs against any user-supplied target sequence. A companion validator module enables targeted verification of user-specified miRNAs against a target sequence by filtering the full SeqFinder prediction output to the requested miRNA identifiers, supporting the same search modes, scoring, and confidence classification as the main module. Both entry points allow users to execute analyses directly from the terminal without requiring web access or external database configuration. Human miRNA data retrieved from miRDB and miRBase, as described above, are bundled within the package to enable offline functionality. The SeqFinder module enables target site prediction with thermodynamic ranking across user-provided mRNA sequences. Installation and basic usage instructions are provided in the GitHub repository documentation (https://github.com/somenath-combio/mirnaprotpred).

### 2.4 Sequence processing and standardisation

miRNAProtPred accepts multiple sequence input formats to accommodate diverse user needs: 3′UTR sequences, coding DNA sequences (CDS), RNA sequences, and protein sequences. Sequence type is automatically detected based on character composition. Sequences containing only A, T, G, C, are treated as DNA; sequences containing U instead of T are classified as RNA; sequences containing standard amino acid single-letter codes are treated as protein. All submitted sequences undergo standardized preprocessing to ensure uniformity. RNA sequences are converted to DNA format by replacing uracil (U) with thymine (T).

For protein sequence inputs, the package attempts to retrieve corresponding nucleotide sequences using NCBI’s tblastn algorithm *via* Biopython’s BLAST and Entrez modules. The retrieved best-matching nucleotide sequence is processed through the same pipeline as direct nucleotide input. Because tblastn performs local alignment against the full NCBI nucleotide database, the retrieved sequence may correspond to an isolate, strain, synthetic construct, or partial CDS that differs at the nucleotide level from the user’s intended reference genome, even when protein-level conservation is high. For proteins encoded by multi-exon spliced mRNAs, tblastn cannot reconstruct the mature spliced coding sequence and may return to a pre-spliced genomic region, which further compounds the divergence. This behavior is inherent to the NCBI BLAST algorithm rather than to miRNAProtPred. Concordance benchmarks demonstrate that the protein-input mode reproduces direct nucleotide input with perfect fidelity when tblastn returns a reference-identical sequence and preserves thermodynamic ranking of shared miRNAs with high fidelity when retrieval returns a divergent sequence, although identity-level miRNA recall degrades in the latter case. Users requiring reference-consistent predictions are therefore advised to provide a verified nucleotide sequence directly. All input sequences must be supplied in the 5′ to 3′ orientation.

Following sequence standardisation, the module employs a sequential prediction pipeline comprising three stages: (i) seed complementarity detection *via* Boyer-Moore string matching, with optional wobble-permissive fallback in relaxed mode; (ii) thermodynamic evaluation of predicted duplexes *via* ViennaRNA RNAduplex, supplemented by flanking AU content assessment; and (iii) multi-criteria confidence classification incorporating seed length, binding energy, AU context, motif identity, and match type.

#### 2.4.1 Stage 1: seed region complementarity matching

The system uses the Boyer-Moore string matching algorithm to detect complementary seed sequences within the processed DNA sequence (Boyer and Moore, 1977). The seed region is defined as nucleotides 2-8 at the 5′ end of the mature miRNA, consistent with canonical miRNA targeting rules. In the default strict mode, the algorithm requires exact Watson-Crick base pairing complementarity (A-U/T and G-C) within the seed region. G:U wobble pairing, single-nucleotide mismatches, and bulged nucleotides within the seed are not permitted. Seeds exhibiting low-complexity characteristics, defined as sequences with fewer than three unique nucleotides or containing homopolymer runs of four or more identical bases, are excluded from the search to reduce spurious matches. Additionally, seeds generating more than 20 matches against the target sequence are flagged as repetitive and excluded, preventing high-frequency motifs from dominating the prediction output. This design represents a deliberate positioning toward the precision end of the precision-recall spectrum, motivated by three considerations. First, canonical seed-matched sites (7mer-m8, 7mer-A1, and 8mer) mediate the large majority of experimentally validated miRNA-target interactions and are the most reliable predictors of functional repression in high-throughput datasets (Lewis et al., 2005; Bartel, 2009; Agarwal et al., 2015). Second, permitting wobble pairs or mismatches within the seed substantially expands the candidate list: in the benchmarking analysis presented in Section 3.7, miRanda’s allowance of G:U wobble and seed-region mismatches increased the number of predicted binding sites by 1.5- to 4.8-fold relative to miRNAProtPred, with the additional candidates predominantly representing lower-confidence interactions that would require extensive experimental follow-up. Third, for cross-species and viral targets where validated training data are sparse, minimizing false positives is often more operationally valuable than maximizing sensitivity, because downstream validation (e.g., luciferase reporter assays, western blots) is resource-intensive and typically limited to 5–20 candidates per study. Consequently, miRNAProtPred prioritizes canonical seed-matched sites in its default strict mode, while acknowledging that biologically functional non-canonical interactions exist and may account for a subset of experimentally validated miRNAs not recovered by strict matching.

miRNAProtPred additionally supports a user-selectable relaxed mode that extends sensitivity to G:U wobble-supported interactions through a hierarchical exact-first strategy. In relaxed mode, for each miRNA seed, exact matches are identified first using the same Boyer-Moore algorithm described above; wobble-permissive variants are generated and searched only when no exact match exists for that seed in the target sequence. This hierarchical design ensures that canonical predictions are never displaced or contaminated by wobble-supported alternatives. Wobble variants are restricted to single G:U substitutions within the seed, reflecting the thermodynamically stable non-Watson-Crick base pair well-documented in RNA duplex structures (Varani and McClain, 2000). G:U wobble base pairs form two hydrogen bonds and represent the most common non-Watson-Crick interaction in RNA helices; their presence at functional miRNA-target interfaces has been documented in both endogenous and cross-species contexts (Doench and Sharp, 2004; Brennecke et al., 2005). Each variant introduces exactly one G-to-U or U-to-G substitution at a single seed position, and the substitution position is recorded in the output. To reflect the reduced confidence associated with non-canonical pairing, wobble-supported interactions are excluded from the High confidence tier regardless of other scoring criteria (see Section 2.4.2), and receive a reduced match-type score in the composite ranking. This ensures that relaxed mode extends sensitivity without inflating the high-confidence canonical prediction set.

The Boyer-Moore algorithm offers computational efficiency with time complexity of O(n + m), where n represents the target sequence length and m represents the seed sequence length, enabling rapid genome-wide scanning (Boyer and Moore, 1977). Following seed match identification, the algorithm records all instances where seed complementarity is detected, along with their precise genomic coordinates within the input sequence. These seed match positions then serve as anchor points for comprehensive thermodynamic analysis of the full miRNA-target interaction.

#### 2.4.2 Stage 2: thermodynamic stability analysis and confidence classification

For each detected seed match, the algorithm extracts an mRNA segment corresponding to the full mature miRNA length from the target sequence. Specifically, the extraction begins at the genomic position where the seed match was identified and extends to capture a region equal in length to the complete mature miRNA sequence (typically 20–24 nucleotides) (Kozomara et al., 2019). This means that if the seed region (nucleotides 2–8 of the miRNA) matched at a particular position, the extracted complementary target site (CTS) includes the nucleotide corresponding to position 1 of the miRNA upstream of the seed match, the seed-matching region itself (positions 2–8), and the remaining downstream nucleotides corresponding to positions 9 through the 3′ end of the miRNA. The minimum free energy (MFE) of duplex formation between the complete mature miRNA sequence and this extracted CTS is then calculated using ViennaRNA’s RNAduplex function. MFE quantifies the thermodynamic stability of the predicted miRNA-mRNA duplex, with more negative values indicating stronger, more stable binding interactions (Rehmsmeier et al., 2004).

In addition to MFE, miRNAProtPred evaluates the AU content of the flanking sequence context surrounding each predicted binding site. For each CTS, nucleotide composition is assessed within a 20-nucleotide window flanking both sides of the binding site, and the proportion of A and U (or T) residues is calculated. AU-rich flanking regions have been established as a significant determinant of functional miRNA targeting efficacy, as they are associated with increased structural accessibility of the target site (Grimson et al., 2007). The motif identity score quantifies the sequence-level concordance between the seed region and the target site within the extracted CTS, providing a measure of alignment precision independent of the thermodynamic calculation.

Predictions are classified into three confidence tiers based on a composite assessment of interaction quality. High confidence requires simultaneous satisfaction of all of the following criteria: seed length ≥7 nucleotides, MFE ≤ −12 kcal/mol, motif identity ≥0.999, AU content in flanking regions ≥0.45, absence of repetitive motif signatures, and exact Watson-Crick seed matching. Medium confidence requires seed length ≥6 nucleotides, MFE between −12 and −10 kcal/mol (inclusive), motif identity ≥0.85, and AU context ≥0.40. All remaining interactions are classified as Low confidence. This multi-criteria framework ensures that the High confidence tier reflects not only thermodynamic stability but also sequence-level interaction quality and local structural accessibility. Critically, wobble-supported interactions identified through relaxed mode are excluded from the High confidence tier regardless of MFE, as the exact-match requirement enforces a principled distinction between canonical and non-canonical seed pairing in the confidence hierarchy.

The −12 kcal/mol threshold for the high confidence MFE criterion is based on evidence from prior miRNA–target prediction and validation studies (Hsu, 2006; Hsu et al., 2011), which indicate that miRNA–mRNA duplexes with MFE values more negative than −12 kcal/mol represent thermodynamically stable interactions with a higher likelihood of functional regulatory activity. This threshold enables prioritization of thermodynamically favorable interactions most likely to produce functional consequences.

miRNAProtPred applies a single set of confidence criteria to all predicted binding sites regardless of target region and does not differentially weight 3′UTR and CDS predictions. This design choice reflects two considerations. First, a unified criterion enables direct comparison of binding-site stability across any submitted sequence context, including viral genomes, synthetic constructs, and cross-species targets for which 3′UTR-specific regulatory rules may not apply. Second, while the endogenous 3′UTR–CDS asymmetry in translational repression is well-documented in mammalian systems (Grimson et al., 2007; Gu et al., 2009; Hausser et al., 2013), its transferability to viral +ssRNAs and other non-canonical contexts remains an active area of investigation (Trobaugh and Klimstra, 2017). Users interpreting miRNAProtPred predictions in endogenous mammalian contexts should therefore apply the well-established prior that 3′UTR sites are more likely to yield functional repression than equivalently scored CDS sites.

### 2.5 Computational validation of the protein input pathway

To evaluate whether the optional protein-to-nucleotide conversion step reproduces predictions generated from direct nucleotide input, concordance analyses were performed for two representative viral proteins that differ in both evolutionary conservation and coding architecture: the SARS-CoV-2 Nucleocapsid phosphoprotein (UniProt P0DTC9; UniProt proteome UP000464024), which is encoded by a single contiguous coding region, and the HIV-1 Rev protein (UniProt Q9Q6W0; UniProt proteome UP000107373), which is encoded by two exons that undergo splicing to generate the mature Rev mRNA. For each protein, the SeqFinder module was executed twice. In the protein-input mode, the amino acid sequence was submitted directly, and the corresponding nucleotide sequence was retrieved through Biopython-based tblastn alignment against the NCBI nucleotide database. In the nucleotide-input mode, the matched coding sequence was extracted from the RefSeq reference genome and supplied directly. For the SARS-CoV-2 Nucleocapsid, nucleotide input comprised NC_045512.2 positions 28,274–29,533 (1,260 bp), corresponding to the single-exon Nucleocapsid coding region. For HIV-1 Rev, nucleotide input comprised the concatenation of Exon 1 (NC_001802.1 positions 5,516–5,591; 76 bp) and Exon 2 (positions 7,925–8,199; 275 bp), yielding a 351 bp mature spliced Rev coding sequence. Predictions from the two input modes were compared on three metrics: (i) the percentage of unique miRNAs identified in both analyses (recall), (ii) the percentage of high-confidence miRNAs (MFE ≤ −12 kcal/mol) identified in both analyses, and (iii) the Pearson correlation coefficient and the mean absolute difference of the top-hit MFE values for miRNAs recovered in both analyses.

### 2.6 Output format

SeqFinder generates comprehensive output containing miRNA description, human miRNA ID, miRBase accession number, full mature miRNA sequence, matched seed sequence, binding position within the input sequence, complementary target site (CTS) sequence, calculated MFE value (kcal/mol), AU context score, and confidence classification (High/Medium/Low). In relaxed mode, the output additionally includes match type (Exact Match or Wobble Pairing), wobble substitution position, and substitution identity, enabling users to distinguish canonical from wobble-supported predictions. Results are automatically sorted by MFE in ascending order (most negative first) to prioritize the strongest predicted interactions.

**FIGURE 1:**
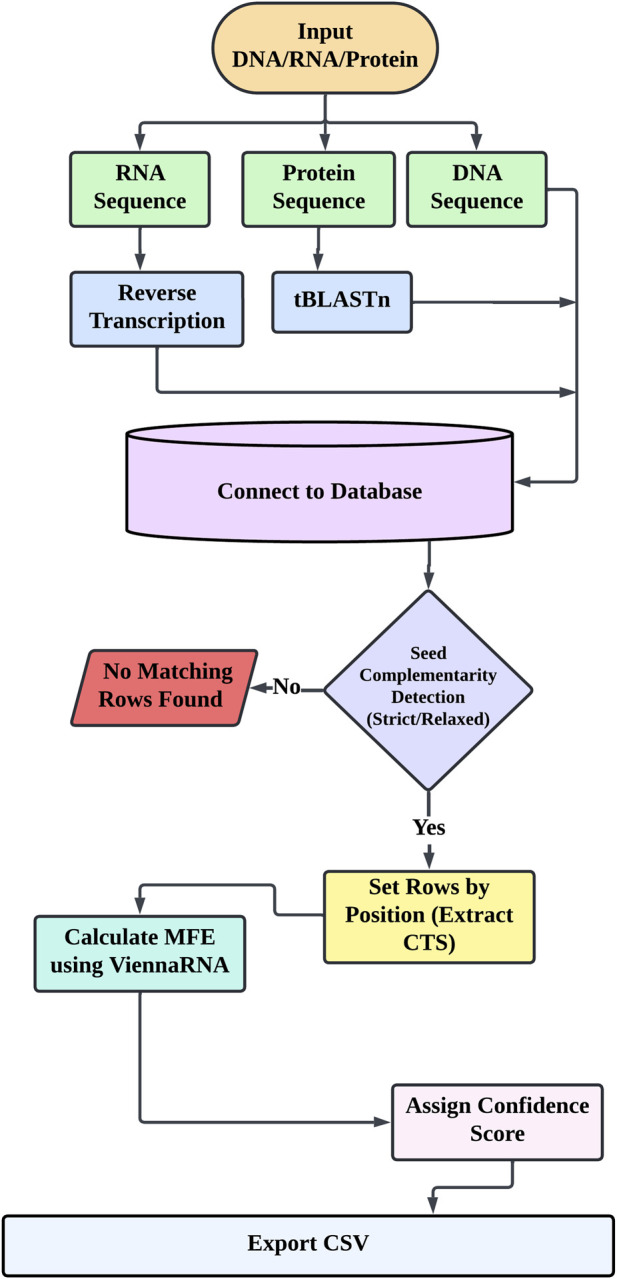
Workflow of miRNAProtPred for human miRNA-target prediction through seed complementarity matching and thermodynamic evaluation.

The package supports four output modes selectable *via* the--output parameter to accommodate different analytical objectives: concise (deduplicated, cluster-representative predictions), raw (all scored interactions with full diagnostic columns), highconf (High confidence predictions only), and highconf_raw (High confidence predictions with full diagnostic columns). Users are prompted to export results to a CSV file for downstream analysis. The overall workflow is illustrated in Figure 1.

### 2.7 Validation strategy and performance assessment

The predictive accuracy of miRNAProtPred was assessed through cross-validation with experimentally validated antiviral miRNAs compiled from published literature. We applied the SeqFinder module to complete viral genome sequences and individual protein coding sequences for both SARS-CoV-2 and HIV-1 using the datasets described before. Predictions for experimentally validated miRNAs were extracted using the companion validator module, which filters genome-wide SeqFinder output to user-specified miRNA identifiers while preserving the same search modes, scoring, and confidence classification. Validation was performed in both strict and relaxed modes to assess the sensitivity contributed by wobble-permissive matching. Predictions were systematically compared against the compiled literature-curated datasets of experimentally validated antiviral miRNAs to quantify recovery rate and sensitivity.

We calculated the proportion of validated antiviral miRNAs successfully identified by miRNAProtPred predictions, providing a direct measure of the tool’s sensitivity for detecting functionally relevant miRNA-pathogen interactions. This comparison enabled assessment of whether miRNAs with established antiviral activity in experimental systems were successfully recovered by our computational approach. For each recovered miRNA, we documented the predicted target regions (3′UTR, coding sequences, or both), binding energies (MFE values), and confidence classifications (high/medium/low) to evaluate the thermodynamic characteristics of experimentally confirmed interactions.

**FIGURE 2:**
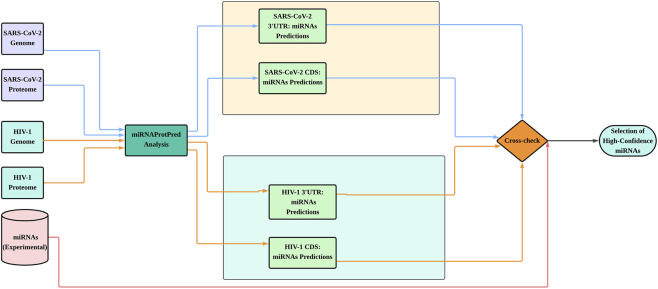
Validation workflow and analysis pipeline for miRNAProtPred.

Additionally, we performed comparative analysis of binding patterns across viral genomes to identify miRNAs with dual-targeting capability, those predicted to bind both 3′UTR and coding sequence regions. These multi-targeting candidates are of particular therapeutic interest as they may simultaneously suppress viral gene expression through multiple regulatory mechanisms. The validation workflow and subsequent comparative analysis framework are summarized in Figure 2.

### 2.8 Comparative benchmarking against sequence-based prediction tools

To evaluate the predictive performance of miRNAProtPred relative to established miRNA target prediction tools, a comparative benchmark was conducted using miRanda v3.3a; (Enright et al., 2003; John et al., 2004); and RNAhybrid v2.1.2 (Kruger and Rehmsmeier, 2006). These tools accept arbitrary miRNA and target RNA sequences without requiring species-specific conservation data, pre-computed UTR annotations, or pre-trained machine-learning models. This enabled direct application to viral genomes while maintaining methodological consistency across comparative analyses. miRanda was executed using a gap open penalty of −9.0, gap extension penalty of −4.0, minimum alignment score threshold of 140, maximum free energy threshold of ≤ −12.0 kcal/mol, and a scaling factor of 4.0. RNAhybrid was executed with default duplex parameters and p-value computation enabled, using the 3utr_human background distribution as a proxy in the absence of a virus-specific statistical model. This represents a necessary approximation, as RNAhybrid’s length-dependent statistical framework is calibrated on human transcriptome characteristics and becomes increasingly conservative for long target sequences. Accordingly, RNAhybrid results were evaluated both at the conventional significance threshold (p ≤ 0.05) and without statistical filtering (raw predictions), to capture its full operating behaviour on viral genomes.

It should be noted that the miRanda and RNAhybrid analyses each required manual preparation of individual miRNA FASTA files, per-miRNA execution against each target sequence, and custom parsing of tool-specific output formats, whereas miRNAProtPred performed the equivalent analysis through a single command-line invocation with no external file preparation, illustrating the workflow-integration difference that the tool is designed to address. Validation was performed using miRNAProtPred in both strict and relaxed modes to assess the contribution of wobble-permissive matching relative to established tools. A feature-level comparison with miRanda and RNAhybrid is provided in Table 1.

**TABLE 1:** Comparative functional analysis of miRNAProtPred, miRanda, and RNAhybrid.

Feature	miRNAProtPred	miRanda	RNAhybrid
Installation	pip install miRNAProtPred	Manual compilation from source	Manual compilation from source
Bundled miRNA database	Yes (2,656 human miRNAs, miRDB + miRBase)	No; user must compile FASTA files	No; user must compile FASTA files
Accepted input formats	DNA, RNA, protein	RNA only (user-prepared)	RNA only (user-prepared)
Automatic sequence type detection	Yes	No	No
Protein-to-nucleotide conversion	Yes (automated tblastn)	No	No
Genome-wide miRNA scanning (single command)	Yes; all 2,656 miRNAs screened per run	No; each miRNA queried individually or *via* custom scripting	No; each miRNA queried individually or *via* custom scripting
Thermodynamic scoring	ViennaRNA RNAduplex (MFE)	Integrated (proprietary scoring + MFE)	Integrated (MFE + p-value)
Wobble-permissive mode	Yes; optional GU wobble-aware seed matching	Limited/internal handling only	Limited/internal handling only
Multi-criteria confidence classification	Yes; integrates seed pairing, MFE, and alignment quality into High/Medium/Low confidence classes	No integrated confidence classification	No integrated confidence classification
Targeted miRNA validation mode	Yes; supports validation against user-specified miRNA sets	Requires manual scripting/workflow customization	Requires manual scripting/workflow customization
Confidence classification	Automatic (High/Medium/Low)	Not provided; user applies *post hoc* thresholds	p-value only; no categorical classification
Structured CSV output with miRBase accessions	Yes	No	No
External database/annotation requirements	None	User must obtain miRNA sequences, format inputs	User must obtain miRNA sequences, format inputs, select background model

The benchmark was designed as a focused validation-based comparison rather than a genome-wide screening exercise, to ensure consistent evaluation across tools with differing internal candidate-generation strategies. The experimentally validated antiviral miRNA sets (16 miRNAs for SARS-CoV-2 and 13 miRNAs for HIV-1) were used as the query set for all tools. Each mature miRNA sequence was submitted individually to miRanda and RNAhybrid against the same viral target sequences used in the miRNAProtPred analysis: the 3′UTR regions of SARS-CoV-2 (NC_045512.2 positions 29,675–29,903) and HIV-1 (NC_001802.1 positions 8,631–9,085), as well as the coding nucleotide sequences of all viral proteins derived from the corresponding reference genomes. This approach ensures that all tools are evaluated on the same validated miRNA-target pairs and allows direct comparison of recall, candidate list size, and regional sensitivity.

An important algorithmic distinction should be considered when interpreting the results. miRNAProtPred in strict mode enforces strict canonical Watson-Crick base pairing (A–U and G–C) within the seed region (positions 2–8), without allowing mismatches or wobble pairing; in relaxed mode, single G:U wobble substitutions are permitted through the hierarchical exact-first fallback strategy described in Section 2.4.1. In contrast, miRanda and RNAhybrid permit mismatches, G:U wobble pairing, and gap formation to varying extents as default behavior. These differences reflect distinct positions along the precision–recall spectrum: miRNAProtPred in strict mode is expected to yield higher precision with more compact candidate sets, whereas the more permissive matching strategies of miRanda and RNAhybrid are expected to increase recall at the cost of larger prediction outputs. The benchmark therefore evaluates how these design choices influence both recovery of validated miRNAs and the size of candidate lists requiring downstream experimental validation. Performance was assessed using four metrics: (i) recall of validated miRNAs, defined as the proportion of the reference set for which at least one binding site was predicted; (ii) total number of predicted binding sites (candidate list size); (iii) mean number of binding sites per recovered miRNA; and (iv) region-specific recovery within 3′UTR and CDS regions, to assess differences in regional sensitivity.

### 2.9 Large-scale classification benchmarking on the miRAW dataset

To evaluate miRNAProtPred’s classification performance beyond virus-specific validation and to provide balanced performance metrics including F1-score, Matthews correlation coefficient (MCC), and area under the receiver operating characteristic curve (ROC-AUC), we benchmarked the tool against the miRAW site-level dataset (Pla et al., 2018). This dataset integrates experimentally validated miRNA-mRNA interactions from DIANA-TarBase v7.0 (Vlachos et al., 2015) and miRTarBase v6.1 (Chou et al., 2018), comprising 62,215 site-level interaction pairs with balanced class distribution (32,075 positive interactions, 51.56%; 30,140 negative interactions, 48.44%). Positive interaction sites were derived from high-throughput experimental evidence including PAR-CLIP, CLASH, and computationally predicted conserved sites from TargetScanHuman 7.1, filtered for thermodynamic plausibility using RNACofold duplex stability calculations. Negative interaction sites were derived from experimentally verified non-targeting miRNA-mRNA pairs, scanned across 3′UTR regions and filtered to retain only thermodynamically plausible but experimentally non-functional binding configurations (Pla et al., 2018). The miRAW dataset has been widely adopted as a standard benchmark in miRNA target prediction studies, including miTAR (Gu et al., 2021), TEC-miTarget (Yang et al., 2024), and miCGR (Wu et al., 2024), enabling direct performance comparison across methods evaluated on the same underlying experimental data.

For each miRNA-target site pair in the dataset, the validator module was executed to determine whether miRNAProtPred predicted a binding interaction. A positive prediction (YES) was recorded when the miRNA was recovered by the SeqFinder engine against the corresponding target site sequence; a negative prediction (NO) was recorded otherwise. Evaluation was performed in both strict and relaxed modes to quantify the precision-recall trade-off across search strategies. Classification performance was assessed using precision, recall (sensitivity), specificity, accuracy, F1-score, MCC, and ROC-AUC. The confusion matrix was constructed from the binary prediction (YES/NO) and the ground-truth interaction label (positive/negative) for all 62,215 pairs. This evaluation provides an independent, unbiased assessment of miRNAProtPred’s predictive performance on endogenous human miRNA-target interactions that were not used in the development or parameterization of the tool.

### 2.10 Usage examples

The miRNAProtPred command-line interface provides two entry points after pip installation: SeqFinder for genome-wide miRNA target discovery, and validator for targeted verification of user-specified miRNAs. Both accept target sequences as either FASTA files or direct nucleotide/protein strings and share the same core parameters: −−mode (strict or relaxed; default: strict), −−output (concise, raw, highconf, or highconf_raw; default: concise), and −−out for automatic CSV export. Complete Installation, documentation, and usage instructions are available at https://mirnaprotpred.biounfold.in/. Representative usage scenarios for both modules are summarized below.

• Example 1. Direct nucleotide input (e.g., viral 3′ UTR).

The SARS-CoV-2 3′UTR nucleotide sequence (5’→3′, DNA or RNA) was provided as a FASTA file to SeqFinder *via* the terminal:

SeqFinder sars_3utr.fasta

This executes the full prediction pipeline in default strict mode with concise output, screening all 2,656 human miRNAs against the target sequence and displaying cluster-representative predictions ranked by composite score, with confidence classification in the final column. To export results directly to CSV:

SeqFinder sars_3utr.fasta−−out sars_3utr_results.csv

Alternatively, the target sequence can be provided as a direct string without a FASTA file:

SeqFinder “AUGCAUGCAUGCAUGC”

• Example 2. Protein input with automated tblastn retrieval.

When only a protein sequence is available, SeqFinder automatically detects the sequence type and retrieves the best-matching nucleotide region *via* NCBI tblastn.

SeqFinder spike_protein.fasta

Users are advised to verify the retrieved nucleotide sequence when reference-consistent predictions are required, particularly for proteins derived from spliced transcripts.

• Example 3. Relaxed mode with wobble − permissive matching.

To extend sensitivity to G:U wobble-supported interactions, SeqFinder is executed with the −−mode relaxed parameter:

SeqFinder sars_3utr.fasta−−mode relaxed−−output raw−−out sars_relaxed.csv

In relaxed mode, wobble-permissive matching activates only for seeds lacking an exact match in the target sequence. The raw output mode provides all scored interactions with full diagnostic columns, including match type (Exact Match or Wobble Pairing), wobble substitution position, and substitution identity.

• Example 4. High‐confidence filtering.

To restrict output to High confidence predictions only:

$$\text{SeqFinder }sars_{genome}.fasta--output\;highconf$$

The highconf mode displays only predictions satisfying all High confidence criteria (seed length ≥7, MFE ≤ −12 kcal/mol, AU context ≥0.45, motif identity ≥0.999, exact match). The highconf_raw mode provides the same filtered set with additional diagnostic columns:

SeqFinder sars_genome.fasta−−output highconf_raw−−out sars_highconf.csv

• Example 5. Targeted miRNA validation.

The companion validator entry point enables verification of specific miRNAs against a target sequence. miRNA identifiers can be provided in three formats: as comma-separated values, as a plain text file (one miRNA ID per line), or as a FASTA file from which miRNA IDs are automatically extracted:

$$validator\;hsa-miR-29a-3p,hsa-miR-138-5p\;sars_{genome}.fasta$$

$$validator\;mirna_{list}.txt\;hiv_{3utr}.fasta--mode\;relaxed$$

$$validator\;known_{mirnas}.fasta\;sars_{genome}.fasta--details$$

The −−details flag displays the full SeqFinder-style interaction output for each recovered miRNA, including binding positions, CTS sequences, MFE values, AU context scores, and confidence classifications. By default, the validator produces a concise YES/NO validation summary. Results can be exported to CSV using the −−out parameter:

validator mirna_list.txt hiv_genome.fasta−−output raw−−out hiv_validation.csv

## 3 Results

### 3.1 Dataset compilation, package development, and prediction analysis

A comprehensive dataset of 2,656 human miRNAs was compiled from miRDB and miRBase databases (Kozomara et al., 2019). Each entry includes complete annotation comprising miRNA identifiers, mature sequences, seed sequences (nucleotides 2–8), and miRBase accession numbers. The complete curated dataset is provided in Supplementary Material S1. Using this dataset, the miRNAProtPred Python package was developed and made freely available for local installation *via* pip. The 3′UTR regions were specifically retrieved for both pathogens, comprising nucleotides 29,675–29,903 (229 bp) for SARS-CoV-2 isolate Wuhan-Hu-1 and nucleotides 8,631–9,085 (455 bp) for HIV-1. To expand the analysis beyond 3′UTR regions, the SeqFinder module was applied to coding sequences of all viral proteins, which were retrieved from UniProt proteome databases, addressing the established phenomenon that miRNAs can inhibit protein translation through binding to coding regions in addition to classical 3′UTR targeting. Details are described in Methods section.

### 3.2 Landscape of predicted interactions for SARS-CoV-2 and HIV-1

**FIGURE 3:**
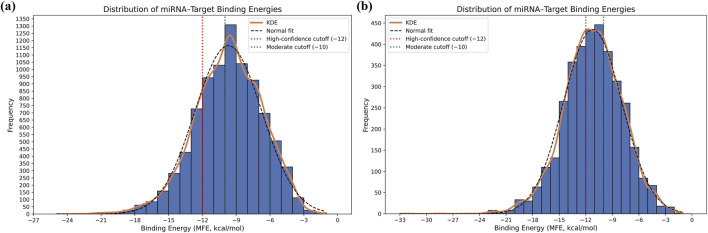
Binding energy distributions for predicted miRNA-viral target interactions. Histograms show frequency distributions of minimum free energy (MFE) values for **(a)** SARS-CoV-2 and **(b)** HIV-1 predictions. Orange curves: kernel density estimation (KDE); black dashed lines: Gaussian fits; red dashed lines: high-confidence cutoff (MFE ≤ −12 kcal/mol); green dashed lines: moderate-confidence cutoff (MFE ≤ −10 kcal/mol) (Hsu, 2006; Hsu et al., 2011).

Application of the seed-based prediction framework in miRNAProtPred yielded 8,719 miRNA–target interactions for SARS-CoV-2, comprising 64 within the 3′UTR and 8,655 across coding sequences. Analysis of binding energy distributions revealed distinct thermodynamic characteristics of the predicted interactions (Figure 3a). Out of the 8,719 total predictions, 1,848 interactions (21.20%) were classified as high-confidence candidates based on minimum free energy (MFE) values ≤ −12 kcal/mol (Hsu, 2006; Hsu et al., 2011), 1,993 interactions (22.86%) fell within the moderate-confidence range (−12 < MFE ≤ −10 kcal/mol), and the remaining 4,878 interactions (55.95%) exhibited low-confidence binding with MFE > −10 kcal/mol. Gaussian fitting of the binding energy distribution yielded a mean MFE of −9.695 ± 2.987 kcal/mol. Kernel density estimation identified a dominant peak at approximately −9.514 kcal/mol, indicating that the majority of predictions fall within the moderate-to low-confidence range. This distribution suggests that while SARS-CoV-2 sequences harbour numerous potential miRNA binding sites, a substantial fraction exhibit moderate thermodynamic stability. Complete SARS-CoV-2 prediction results are provided in Supplementary Material S2.

In purely seed-based process, miRNAProtPred generated 3,587 predictions for HIV-1, with 205 targeting the 3′UTR and 3,382 targeting coding sequences. Thermodynamic analysis of HIV-1 predictions revealed a favorable binding energy profile (Figure 3b). Of the 3,587 total predictions, 1,285 interactions (35.82%) were classified as high-confidence candidates with MFE ≤ −12 kcal/mol, 846 interactions (23.58%) fell within the moderate-confidence range (−12 < MFE ≤ −10 kcal/mol), and 1456 interactions (40.59%) exhibited low-confidence binding with MFE > −10 kcal/mol. Gaussian fitting of the binding energy distribution yielded a mean MFE of −11.215 ± 3.293 kcal/mol. Kernel density estimation identified a dominant peak at approximately −11.600 kcal/mol, demonstrating a strong enrichment for thermodynamically stable interactions. The higher proportion of high-confidence predictions relative to SARS-CoV-2 (35.82% vs. 21.20%) indicates that HIV-1 genomic sequences contain comparatively more thermodynamically favorable miRNA binding sites, consistent with the extensively documented role of host miRNAs in regulating HIV-1 latency and replication. Complete HIV-1 prediction results are provided in Supplementary Material S3.

#### 3.2.1 Region-stratified analysis

**FIGURE 4:**
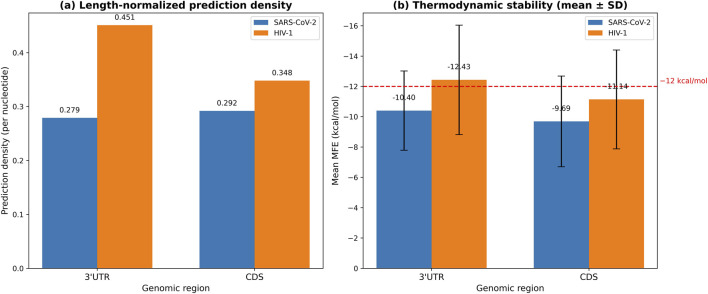
Region-stratified comparison of miRNA-binding predictions between SARS-CoV-2 and HIV-1. **(a)** Length-normalized prediction density reveals a modest enrichment of predicted miRNA-binding sites within the CDS of SARS-CoV-2, whereas HIV-1 shows comparatively higher prediction density in the 3′UTR. **(b)** Mean thermodynamic stability (MFE ±SD) indicates relatively similar binding energies across SARS-CoV-2 regions, while the HIV-1 3′UTR exhibits the strongest average binding stability. Only the HIV-1 3′UTR surpasses the high-confidence threshold (−12 kcal/mol), suggesting region-specific enrichment of stable miRNA interactions in HIV-1 that is less evident in SARS-CoV-2. Overall, these findings indicate that viral genomic organization may influence miRNA-binding distribution and stability.

To determine whether the observed disparity in 3′UTR and CDS prediction counts reflects sequence-length effects alone or also a region-specific thermodynamic asymmetry, MFE distributions and prediction densities were analyzed separately for each genomic region in both viruses (Figure 4). For SARS-CoV-2, the 229 bp 3′UTR yielded 64 predictions at a density of 0.279 predictions per nucleotide, with a mean MFE of −10.40 ± 2.61 kcal/mol, whereas the 29,674 bp combined CDS yielded 8,655 predictions at a density of 0.292 predictions per nucleotide, with a mean MFE of −9.69 ± 2.99 kcal/mol. The thermodynamic distributions of the two regions were therefore nearly indistinguishable (ΔMFE = 0.71 kcal/mol), and the ∼136-fold difference in absolute prediction counts corresponds closely to the ∼129.58-fold length ratio, indicating that the count disparity is driven overwhelmingly by sequence-space effects rather than by a mechanistic preference for CDS targeting. For HIV-1, a different pattern emerged: the 455 bp 3′UTR yielded 205 predictions at a density of 0.451 predictions per nucleotide, with a mean MFE of −12.43 ± 3.61 kcal/mol, whereas the 9,719 bp combined CDS yielded 3,382 predictions at a density of 0.348 predictions per nucleotide, with a mean MFE of −11.14 ± 3.26 kcal/mol. The HIV-1 3′UTR thus exhibited both a 1.29-fold higher prediction density and a 1.29 kcal/mol shift toward more stable binding compared to the HIV-1 CDS, indicating genuine region-specific enrichment for thermodynamically favorable miRNA binding beyond what length alone would predict. This enrichment is consistent with the extensive regulatory architecture of the HIV-1 3′UTR region and supports the biological significance of 3′UTR-mediated miRNA regulation in HIV-1 (Nathans et al., 2009; Houzet and Jeang, 2011). The contrast between the two viruses, length-dominated for SARS-CoV-2 and region-enriched for HIV-1, illustrates that the interpretation of prediction-count disparities between 3′UTR and CDS regions must be virus-specific and cannot be generalized from a single genomic architecture.

#### 3.2.2 Concordance of protein-route and CDS-route predictions

The reliability of the optional protein-input pathway was assessed by comparing predictions obtained from amino acid input against those obtained from the matched reference nucleotide sequence, using two representative viral proteins that differ in evolutionary conservation and in coding architecture.

**FIGURE 5:**
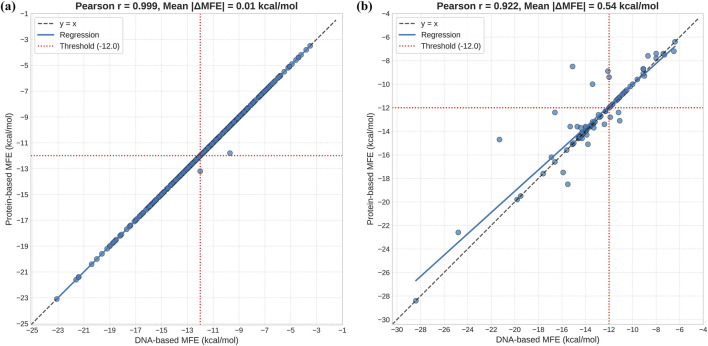
Concordance of top-hit MFE values between protein-input and nucleotide-input modes for **(a)** SARS-CoV-2 Nucleocapsid (P0DTC9) and **(b)** HIV-1 Rev (Q9Q6W0). Each point represents one miRNA recovered by both input modes. Dashed diagonal: y = x; red dotted lines: high-confidence threshold (−12 kcal/mol).

For the SARS-CoV-2 Nucleocapsid phosphoprotein (P0DTC9), the automated tblastn retrieval returned a nucleotide sequence identical to the NC_045512.2 reference across the annotated Nucleocapsid coding region (positions 28,274–29,533). Comparative analysis demonstrated an exceptionally high level of concordance between the two input modes. A total of 485 predictions were identified from the nucleotide-based dataset and 481 from the protein-based dataset, with 395 shared unique miRNAs corresponding to a recall of 100% and a Jaccard similarity index of 99.0%. Both datasets produced an identical number of high-confidence miRNAs (154; MFE ≤ −12 kcal/mol), resulting in a high-confidence recall of 100%. Furthermore, the thermodynamic stability of shared predictions remained highly consistent, with a mean absolute MFE difference of only 0.01 ± 0.12 kcal/mol. Correlation analysis of shared miRNA MFE values revealed an almost perfect agreement between the two approaches, with a Pearson correlation coefficient of 0.999 and a highly significant p-value (0.00e+00) (Figure 5a).

For HIV-1 Rev (Q9Q6W0), the concordance analysis was more challenging for two reasons that reflect the biological complexity of the Rev mRNA rather than the limitation of the miRNAProtPred algorithm itself. First, HIV-1 exhibits substantially greater isolate-level sequence heterogeneity in the NCBI nucleotide database than SARS-CoV-2, and tblastn retrieval consequently returned a nucleotide sequence carrying single-nucleotide substitutions relative to the NC_001802.1 reference. Second, Rev is encoded by two exons (NC_001802.1 positions 5,516–5,591 and 7,925–8,199) that are separated by a ∼2.3 kb intron and joined by splicing to yield the 351 bp mature mRNA used as nucleotide input in this study. Because tblastn returns contiguous alignments, the retrieved sequence may correspond to a pre-spliced genomic region, a separately deposited spliced mRNA, or a partial isolate CDS, each of which introduces divergence beyond simple isolate-level variation. Under these conditions, the protein-based dataset generated 150 predictions compared with 140 predictions from the direct nucleotide-based dataset, with 93 shared unique miRNAs corresponding to a recall of 72.09% and a Jaccard similarity index of 52.84%. High-confidence predictions (MFE ≤ −12 kcal/mol) also showed partial conservation, with 58 shared high-confidence miRNAs and a high-confidence recall of 76.32%. Despite these differences in miRNA composition, thermodynamic agreement among shared predictions remained strong. Shared miRNAs exhibited a Pearson correlation coefficient of 0.922 with a highly significant p-value (2.86 × 10^−39^), while the mean absolute MFE difference remained relatively small at 0.54 ± 1.23 kcal/mol (Figure 5b). The subset of miRNAs uniquely identified in the protein-derived dataset likely reflects sequence differences introduced during tblastn retrieval, including isolate-specific substitutions or transcript structural variation, which can alter canonical seed-match positions and thereby influence downstream miRNA prediction outcomes.

Together, these two cases delimit the operating range of the protein-input mode. When tblastn retrieval returns a sequence identical to the intended reference, as for the single-exon SARS-CoV-2 Nucleocapsid CDS, predictions generated from protein input are indistinguishable from those generated from direct nucleotide input. When tblastn retrieval returns a sequence that diverges from the intended reference, whether through isolate-level variation, splicing architecture, or database heterogeneity, thermodynamic ranking of shared miRNAs is preserved with high fidelity, whereas identity-level recall degrades approximately in proportion to the underlying nucleotide divergence. These observations indicate that the principal determinant of protein-input performance is the fidelity of the tblastn retrieval step rather than the downstream seed-matching or thermodynamic evaluation algorithms. Complete per-miRNA comparisons for both test proteins are provided in Supplementary Material S4.

### 3.3 Prediction on the binding sites of the miRNAs with high-confidence probability

Comprehensive analysis of 1,878 high-confidence miRNA–viral interactions, representing 1,053 unique miRNAs, was performed across both the 3′UTR and the coding regions of all major SARS-CoV-2 proteins, including the ORF1ab polyprotein, Spike glycoprotein, Nucleoprotein, Membrane protein, Envelope protein, and accessory proteins ORF3a, ORF6, ORF7a, ORF7b, ORF8, and ORF10. All interactions reported in this analysis fell within the high-confidence prediction range and displayed distinct non-uniform targeting patterns across the viral genome. The ORF1ab polyprotein accounted for the largest number of high-confidence interactions, with 1,227 predictions involving 800 unique miRNAs. This was followed by the Spike glycoprotein, which exhibited 259 high-confidence interactions involving 218 unique miRNAs, and the Nucleoprotein, which yielded 184 interactions corresponding to 154 unique miRNAs. Additional viral proteins showed comparatively fewer high-confidence predicted interactions, including the Membrane protein with 87 interactions, ORF3a with 47 interactions, ORF7a with 27 interactions, ORF8 with 18 interactions, and the Envelope protein with nine interactions. ORF6, ORF7b, and ORF10 yielded a single high-confidence predicted interaction each. Despite its regulatory importance, the SARS-CoV-2 3′UTR accounted for only 17 high-confidence interactions, likely reflecting its relatively short length of 229 bp.

To place these findings in a broader viral context, the same high-confidence prediction framework was subsequently applied to HIV-1. High-confidence miRNA binding site predictions in HIV-1 identified 1,453 interactions corresponding to 770 unique miRNAs distributed across both the viral 3′UTR and coding regions. Predicted targets included the structural polyproteins Gag and Gag-Pol, the Envelope glycoprotein, and multiple regulatory and accessory proteins involved in viral replication, transcriptional regulation, and immune evasion. The overall distribution revealed extensive miRNA accessibility across both structural and regulatory components of the HIV-1 genome. The Gag-Pol polyprotein emerged as the most frequently targeted region, with 379 high-confidence interactions involving 309 unique miRNAs, followed by the Gag polyprotein with 301 interactions involving 257 unique miRNAs and the Envelope glycoprotein with 265 interactions corresponding to 232 unique miRNAs. Additional regulatory and accessory proteins displayed distinct but comparatively lower targeting densities, including Nef with 88 interactions, Rev with 86 interactions, Tat with 74 interactions, Vif (virion infectivity factor) with 71 interactions, Vpr with 50 interactions, and Vpu with 35 interactions. Notably, the HIV-1 3′UTR accounted for 104 high-confidence interactions involving 97 unique miRNAs, underscoring the continued importance of this regulatory region in miRNA-mediated modulation of viral gene expression and latency.

### 3.4 Cross-validation with experimentally validated antiviral miRNAs

To validate the miRNAProtPred’s predictive accuracy, its capacity to identify miRNAs that had been experimentally confirmed as viral inhibitors was analysed based on the assumption that the inhibitors can bind to the viral genomic mRNAs.

For SARS-CoV-2, Vaddadi et al. (2023) tested 39 computationally predicted miRNAs against live SARS-CoV-2 virus experimentally and demonstrated that seven miRNAs, hsa-miR-15a-5p, hsa-miR-153-3p, hsa-miR-298, hsa-miR-497, hsa-miR-508-3p, hsa-miR-1909-3p, and hsa-miR-3130-3p, reduced spike protein levels by more than 50% in Western blot analysis (Vaddadi et al., 2023). Among these, six miRNAs also significantly inhibited viral replication, while hsa-miR-497 reduced spike protein expression without significantly affecting viral replication. miRNAProtPred successfully identified all seven inhibitors, with five classified as high-confidence (MFE ≤ −12 kcal/mol) and two as medium-confidence candidates. Further experimental support comes from Siniscalchi et al. (2021), who validated hsa-miR-29a-3p binding to SARS-CoV-2 sequences through reporter-based assays (Siniscalchi et al., 2021), and Wang et al. (2021), who demonstrated that four exosomal miRNAs; hsa-miR-7-5p, hsa-miR-24-3p, hsa-miR-145-5p, and hsa-miR-223-3p; directly inhibit spike protein expression and viral replication (Wang et al., 2021). miRNAProtPred successfully predicted all four exosomal miRNAs. hsa-miR-29a-3p and hsa-miR-24-3p were classified as high confidence, hsa-miR-7-5p as high confidence with predicted binding sites in both the polyprotein and Spike coding regions, hsa-miR-145-5p as high confidence with binding sites distributed across the polyprotein, Spike, and Membrane coding regions, and hsa-miR-223-3p as medium confidence with predicted binding in the polyprotein coding region (Table 2).

**TABLE 2:** Experimentally validated inhibitors of SARS-CoV-2 and HIV-1.

SARS-CoV-2			
miRNA	Reported effect (Reference)	Predicted interaction (energy)	Mode of prediction
hsa-miR-15a-5p	Reduces spike expression (Vaddadi et al., 2023)	15 (−16.6 kcal/mol)	Strict
hsa-miR-153-3p	Reduces spike expression (Vaddadi et al., 2023)	7 (−11.0 kcal/mol)	Strict
hsa-miR-298	Suppresses spike protein (Vaddadi et al., 2023)	6 (−16.3 kcal/mol)	Strict
hsa-miR-497-5p	Reduces spike expression (Vaddadi et al., 2023)	15 (−15.3 kcal/mol)	Strict
hsa-miR-508-3p	Reduces spike expression (Vaddadi et al., 2023)	6 (−11.0 kcal/mol)	Strict
hsa-miR-1909-3p	Reduces spike expression (Vaddadi et al., 2023)	2 (−15.3 kcal/mol)	Strict
hsa-miR-3130-3p	Reduces spike expression (Vaddadi et al., 2023)	4 (−14.8 kcal/mol)	Strict
hsa-miR-29a-3p	Validated viral binding (Siniscalchi et al., 2021)	12 (−13.4 kcal/mol)	Strict
hsa-miR-3941	Validated viral binding (Barreda-Manso et al., 2021)	7 (−10.9 kcal/mol)	Strict
hsa-miR-128-1-5p	Validated viral binding (Barreda-Manso et al., 2021)	2 (−17.1 kcal/mol)	Strict
hsa-miR-138-5p	Validated viral binding (Barreda-Manso et al., 2021)	3 (−14.6 kcal/mol)	Strict
hsa-miR-365b-5p	Validated viral binding (Barreda-Manso et al., 2021)	1 (−13.3 kcal/mol)	Strict
hsa-miR-7-5p	Exosomal viral inhibition (Wang et al., 2021)	2 (−12.8 kcal/mol)	Strict
hsa-miR-24-3p	Exosomal viral inhibition (Wang et al., 2021)	2 (−14.3 kcal/mol)	Strict
hsa-miR-145-5p	Exosomal viral inhibition (Wang et al., 2021)	10 (−14.0 kcal/mol)	Strict
hsa-miR-223-3p	Exosomal viral inhibition (Wang et al., 2021)	3 (−10.0 kcal/mol)	Strict

HIV-1			
miRNA	Reported effect (Reference)	Predicted interaction (energy)	Mode of prediction
hsa-miR-138-5p	Antiviral activity (Houzet et al., 2012)	6 (−14.6 kcal/mol)	Strict
hsa-miR-149-5p	Suppresses replication (Houzet et al., 2012)	4 (−17.0 kcal/mol)	Strict
hsa-miR-92a-3p	Reduces viral expression (Houzet et al., 2012)	2 (−11.8 kcal/mol)	Relaxed^a^
hsa-miR-28-5p	Maintains viral latency (Huang et al., 2007)	4 (−16.0 kcal/mol)	Strict
hsa-miR-150-5p	Restricts HIV-1 transcription (Huang et al., 2007)	2 (−11.8 kcal/mol)	Strict
hsa-miR-223-3p	Latency-associated inhibition (Huang et al., 2007)	2 (−11.2 kcal/mol)	Strict
hsa-miR-155-5p	Direct antiviral activity (Swaminathan et al., 2012)	5 (−9.1 kcal/mol)	Strict
hsa-miR-29a-3p	Direct replication inhibition (Nathans et al., 2009)	2 (−13.4 kcal/mol)	Strict
hsa-miR-29b-3p	Suppresses replication (Nathans et al., 2009)	2 (−13.4 kcal/mol)	Strict
hsa-miR-29c-3p	Suppresses replication (Nathans et al., 2009)	2 (−13.4 kcal/mol)	Strict
hsa-miR-1290	Contributes to HIV-1 latency (Wang et al., 2015)	3 (−10.5 kcal/mol)	Strict
hsa-miR-125b-5p	Maintains viral latency (Huang et al., 2007)	2 (−14.1 kcal/mol)	Strict
hsa-miR-382-5p	Maintains viral latency (Huang et al., 2007)	1 (−6.7 kcal/mol)	Relaxed^a^

^a^ Recovered through wobble-permissive matching in relaxed mode; not recovered in strict mode.

Beyond these three datasets, Barreda-Manso et al. (2021) provided independent experimental validation of four human miRNAs targeting the SARS-CoV-2 3′UTR through dual-luciferase reporter assays in HEK293T cells. In their study, mimics of hsa-miR-3941, hsa-miR-128-1-5p, hsa-miR-365b-5p, and hsa-miR-138-5p each produced statistically significant reductions (33%–42%) in luciferase activity driven by the full SARS-CoV-2 3′UTR, confirming direct binding and post-transcriptional repression at this regulatory region (Barreda-Manso et al., 2021). miRNAProtPred recovered all four miRNAs through canonical seed-based prediction. hsa-miR-3941 was predicted to bind the 3′UTR and coding sequence regions, with binding sites in the medium confidence range (MFE = −10.4 kcal/mol in the 3′UTR). The 3′UTR prediction represents a direct computational concordance with the experimental validation, while the additional CDS predictions suggest potential dual-region targeting that may enhance antiviral efficacy through coordinated suppression at multiple viral genomic loci. hsa-miR-128-1-5p was classified as a high confidence candidate with the strongest single predicted binding energy in the entire SARS-CoV-2 validated set (MFE = −17.1 kcal/mol), targeting the Nucleoprotein coding region. hsa-miR-138-5p was also classified as high confidence, with predicted binding sites distributed across the polyprotein and Spike coding regions (MFE = −14.6 and −13.2 kcal/mol). hsa-miR-365b-5p was classified as high confidence with a predicted binding site in the Spike protein coding region (MFE = −13.3 kcal/mol). For hsa-miR-128-1-5p, hsa-miR-138-5p, and hsa-miR-365b-5p, the experimental validation confirmed 3′UTR binding, whereas miRNAProtPred’s predictions mapped to coding sequence regions, illustrating that a single miRNA may engage multiple target regions within the same viral genome and that 3′UTR-validated binding does not preclude additional CDS-level interactions detectable by seed-based scanning.

**FIGURE 6:**
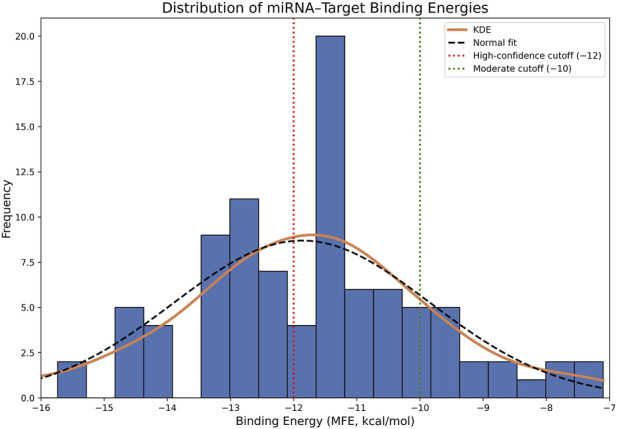
Binding energy distributions of experimentally validated miRNA-viral inhibitors of SARS-CoV-2. Orange curves: kernel density estimation (KDE); black dashed lines: Gaussian fits; red dashed lines: high-confidence cutoff (MFE ≤ −12 kcal/mol); green dashed lines: moderate-confidence cutoff (MFE ≤ −10 kcal/mol).

In total, miRNAProtPred successfully recovered all 16 experimentally supported SARS-CoV-2 inhibitory miRNAs through canonical seed matching in strict mode (100% recovery rate), spanning four independent experimental studies (Barreda-Manso et al., 2021; Siniscalchi et al., 2021; Vaddadi et al., 2023; Wang et al., 2021). While the initial identification of these candidates is influenced by seed-sequence complementarity, further evaluation at the binding-site level enabled assessment of prediction confidence based on the multi-criteria framework. Across the 16 recovered candidates, a total of 97 predicted binding sites were identified, allowing confidence classification at the miRNA level. Twelve miRNAs (75%) were classified as high confidence candidates (MFE ≤ −12 kcal/mol with full multi-criteria satisfaction), whereas four miRNAs (25%) exhibited medium confidence predictions (−12 < MFE ≤ −10 kcal/mol). No validated miRNA fell into the low confidence category. This distribution indicates that the majority of experimentally supported antiviral miRNAs are predicted to form thermodynamically stable duplexes with the SARS-CoV-2 genome, reinforcing the effectiveness of miRNAProtPred’s confidence-driven ranking strategy. Kernel density estimation further revealed a dominant peak near −11.69 kcal/mol, consistent with enrichment for stable miRNA-viral RNA interactions. A detailed summary of all SARS-CoV-2-associated miRNAs, their predicted binding energies, target regions, and regulatory status is provided in Table 2. Analysis of binding energy distributions for the 16 successfully recovered SARS-CoV-2 inhibitory miRNAs confirmed thermodynamic enrichment (Figure 6). Predicted binding energies ranged from −17.1 to −7.1 kcal/mol, with a mean value of −11.87 ± 2.01 kcal/mol, representing a 2.18 kcal/mol shift toward stronger binding compared to the genome-wide prediction landscape (mean = −9.695 kcal/mol).

For HIV-1, host miRNA-mediated restriction has been extensively characterized, with multiple independent studies establishing specific miRNAs as direct or indirect inhibitors of viral replication, transcription, and latency maintenance. Among these, the hsa-miR-29 family represents the most comprehensively validated antiviral miRNA system. Nathans et al. (2009) demonstrated that hsa-miR-29a directly targets the HIV-1 3′UTR, promoting viral RNA sequestration within RISC and P-body complexes and thereby suppressing viral production and infectivity (Nathans et al., 2009). Functional perturbation experiments further showed that inhibition of hsa-miR-29a enhances HIV-1 replication, whereas hsa-miR-29 mimics significantly reduce viral output in human T lymphocytes. Consistent with these findings, miRNAProtPred successfully identified all three family members (hsa-miR-29a-3p, hsa-miR-29b-3p, and hsa-miR-29c-3p) as high confidence HIV-1-targeting miRNAs, each exhibiting dual-targeting of the 3′UTR and Nef coding region with MFE values of −13.4 kcal/mol for Nef and −13.1 kcal/mol for the 3′UTR. Beyond the hsa-miR-29 family, miRNAProtPred accurately identified a broader repertoire of experimentally validated HIV-1 inhibitory miRNAs. Notably, miRNAProtPred successfully predicted hsa-miR-1290, which Wang et al. (2015) showed contributes to HIV-1 latency (Wang et al., 2015), as well as hsa-miR-138-5p and hsa-miR-149-5p reported by Houzet et al. (2012) as antiviral miRNAs with sequence complementarity correlating with restriction potency (Houzet et al., 2012). A third miRNA from the Houzet study, hsa-miR-92a-3p, was not recovered in strict mode and is addressed through the relaxed mode analysis described below. In addition, miRNAProtPred recovered hsa-miR-155-5p, which Swaminathan et al. (2012) demonstrated exerts direct antiviral activity through targeting host dependency factors required for viral pre-integration (Swaminathan et al., 2012). A landmark study by Huang et al. (2007) further established that five miRNAs, hsa-miR-28-5p, hsa-miR-125b-5p, hsa-miR-150-5p, hsa-miR-223-3p, and hsa-miR-382-5p, are enriched in resting CD4^+^ T cells and contribute to HIV-1 latency through targeting the 3′ ends of viral mRNAs (Huang et al., 2007). miRNAProtPred in strict mode recovered four of these five miRNAs; hsa-miR-382-5p was not recovered in strict mode and is addressed through the relaxed mode analysis below. Consistent with these observations, miRNAProtPred classified seven miRNAs as high confidence candidates, including those associated with viral latency maintenance (hsa-miR-28-5p, hsa-miR-125b-5p; Huang et al., 2007), direct suppression of viral gene expression or replication (hsa-miR-138-5p, hsa-miR-149-5p; Houzet et al., 2012), and 3′UTR-mediated viral restriction (hsa-miR-29a-3p, hsa-miR-29b-3p, hsa-miR-29c-3p; Nathans et al., 2009). An additional three miRNAs were classified as medium confidence candidates (hsa-miR-150-5p, hsa-miR-1290, and hsa-miR-223-3p), while one miRNA fell into the low confidence category (hsa-miR-155-5p). Despite its lower predicted binding strength (best MFE = −9.1 kcal/mol), the experimental validation of hsa-miR-155-5p indicates that biologically relevant interactions can occur at weaker binding energies, potentially mediated by cooperative binding mechanisms or cellular context-dependent factors.

In total, 11 of the 13 experimentally validated HIV-1 inhibitory miRNAs were successfully recovered by miRNAProtPred in strict mode, corresponding to 84.6% recovery based on canonical seed-sequence complementarity. The two unrecovered miRNAs, hsa-miR-92a-3p and hsa-miR-382-5p, are addressed through the relaxed mode analysis described below. Across the 11 validated HIV-1 miRNAs recovered in strict mode, a total of 34 predicted binding sites were identified, enabling confidence assessment beyond seed matching alone. At the miRNA level, seven of the eleven predicted miRNAs (63.6%) were classified as high confidence candidates (MFE ≤ −12 kcal/mol with full multi-criteria satisfaction), three miRNAs (27.3%) exhibited medium confidence predictions, and one miRNA (9.1%) was classified as low confidence. A single integrated table summarizing all experimentally validated SARS-CoV-2 and HIV-1 miRNAs, their predicted binding energies, target regions, and regulatory status is provided in Table 2.

**FIGURE 7:**
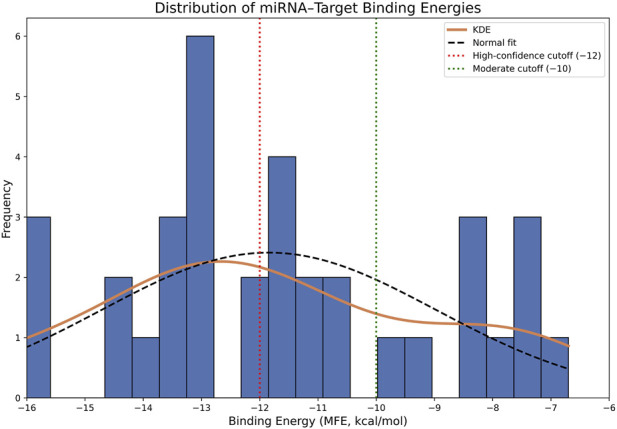
Binding energy distributions of experimentally validated miRNA-viral inhibitors of HIV-1. Orange curves: kernel density estimation (KDE); black dashed lines: Gaussian fits; red dashed lines: high-confidence cutoff (MFE ≤ −12 kcal/mol); green dashed lines: moderate-confidence cutoff (MFE ≤ −10 kcal/mol).

Analysis of binding energy distributions for the validated HIV-1 miRNAs recovered in strict mode revealed thermodynamic enrichment (Figure 7), with predicted binding energies ranging from −17.0 to −7.2 kcal/mol and a mean value of −12.13 ± 2.69 kcal/mol. This represents a 0.92 kcal/mol shift toward stronger binding compared to the genome-wide prediction landscape (mean = −11.215 kcal/mol). Gaussian fitting yielded a mean of −12.13 kcal/mol with a standard deviation of 2.69 kcal/mol, while kernel density estimation identified a dominant peak at approximately −12.80 kcal/mol, indicating enrichment for thermodynamically stable miRNA-viral RNA interactions. The smaller magnitude of thermodynamic enrichment for HIV-1 relative to SARS-CoV-2 (0.92 vs. 2.18 kcal/mol) reflects the higher baseline thermodynamic stability of the HIV-1 genome-wide prediction landscape (mean MFE = −11.215 kcal/mol vs. −9.695 kcal/mol for SARS-CoV-2), which narrows the separation between validated and background distributions. Together with the SARS-CoV-2 validation results, these findings demonstrate that miRNAProtPred reliably identifies experimentally supported antiviral miRNAs at the sequence-recognition level, while enabling further prioritization based on thermodynamic stability.

To evaluate the sensitivity extension provided by wobble-permissive matching, the SeqFinder module was additionally executed in relaxed mode against both viral genomes. For SARS-CoV-2, strict mode alone achieved complete recovery of all 16 validated miRNAs; relaxed mode identified no additional validated miRNAs beyond those recovered by canonical matching, indicating that all validated interactions in this system are accessible through canonical seed complementarity. For HIV-1, the two unrecovered miRNAs were identified through relaxed mode. hsa-miR-92a-3p was recovered *via* a G:U wobble substitution at seed position 6, with predicted binding sites in the Gag-Pol polyprotein (MFE = −11.8 kcal/mol, Medium confidence) and Vpr (MFE = −7.3 kcal/mol, Low confidence). The Gag-Pol interaction represents a thermodynamically plausible binding event accessible only through non-canonical pairing, demonstrating that the relaxed mode can recover biologically meaningful interactions beyond the strict seed-matching boundary. hsa-miR-382-5p was recovered *via* a G:U wobble substitution at seed position 7, targeting the Vpr coding region with a predicted duplex MFE of −6.7 kcal/mol (Low confidence). The weak thermodynamic stability of the hsa-miR-382-5p prediction is notable in the context of the experimental literature: Huang et al. (2007) demonstrated that hsa-miR-382 contributes to HIV-1 latency in resting CD4^+^ T cells, but the mechanism involves cooperative activity with other miRNAs enriched in resting T cells (hsa-miR-28, hsa-miR-125b, hsa-miR-150, hsa-miR-223) rather than independent high-affinity canonical targeting. The marginal binding energy predicted by miRNAProtPred is consistent with this cooperative model. In both cases, the confidence classification framework appropriately distinguished wobble-dependent interactions from canonical predictions, with hsa-miR-92a-3p classified as Medium and hsa-miR-382-5p as Low confidence, preserving the discriminatory value of the tiered scoring system. Combined strict and relaxed analysis therefore achieved complete recovery (13/13) for HIV-1 and (16/16) for SARS-CoV-2.

Overall, analysis of 29 experimentally supported antiviral miRNAs spanning two evolutionarily distinct viral families, SARS-CoV-2 (16 miRNAs) and HIV-1 (13 miRNAs), demonstrated consistent enrichment for thermodynamically stable binding interactions relative to genome-wide prediction landscapes. Of these, 27 miRNAs were successfully recovered through canonical seed-sequence complementarity in strict mode, and the remaining two miRNAs were recovered through wobble-permissive matching in relaxed mode, achieving complete recovery across both viral systems. Importantly, 19 of the 27 strict-mode recovered miRNAs were classified as high confidence candidates, supported by energetically favorable binding interactions and full multi-criteria satisfaction, highlighting the added discriminatory power of the composite confidence framework beyond seed matching alone.

### 3.5 Prediction on the binding sites of the experimentally validated anti-viral miRNAs

To investigate the genomic binding patterns of experimentally validated antiviral miRNAs, we performed a detailed analysis of high-confidence predicted target site locations across both viral genomes. Focusing on the 16 experimentally validated SARS-CoV-2 inhibitory miRNAs and 11 HIV-1 inhibitory miRNAs recovered in strict mode, we restricted the analysis to high-confidence predictions (MFE ≤ −12 kcal/mol with full multi-criteria satisfaction). This filtering yielded 46 high-confidence SARS-CoV-2 binding sites corresponding to 12 unique miRNAs, and 19 high-confidence HIV-1 binding sites corresponding to 7 unique miRNAs, which were subsequently used for binding-site-level characterization.

**FIGURE 8:**
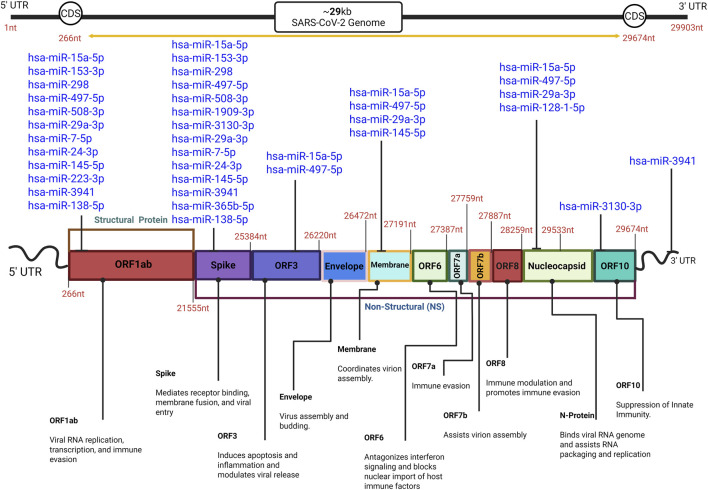
Predicted miRNA binding site distribution across SARS-CoV-2 coding regions.

Analysis of predicted binding positions within the SARS-CoV-2 genome revealed distinct targeting preferences across viral proteins (Figure 8). hsa-miR-15a-5p and hsa-miR-29a-3p exhibited the broadest targeting capability, each with high-confidence binding sites spanning four viral regions: hsa-miR-15a-5p targeted the polyprotein, Spike, Nucleoprotein, and ORF3a, while hsa-miR-29a-3p targeted the polyprotein, Spike, Nucleoprotein, and Membrane protein. hsa-miR-497-5p demonstrated high-confidence binding across three regions (polyprotein, Nucleoprotein, and ORF3a), including the strongest ORF3a interaction in the validated set (MFE = −14.7 kcal/mol). hsa-miR-128-1-5p exhibited the strongest overall predicted binding affinity (MFE = −17.1 kcal/mol) targeting the Nucleoprotein coding region. Among Spike-targeting interactions, hsa-miR-298 showed the strongest binding energy (MFE = −16.3 kcal/mol), followed by hsa-miR-1909-3p (MFE = −15.3 kcal/mol). Target region distribution revealed that the polyprotein was the predominant target, followed by the Spike protein. Within this filtered high-confidence subset, all recovered binding sites for the experimentally validated SARS-CoV-2 inhibitory miRNAs mapped to coding sequences, with no 3′UTR sites passing the high-confidence threshold. Three considerations temper the interpretation of this observation. First, the SARS-CoV-2 3′UTR spans only 229 nucleotides and therefore provides a substantially smaller sequence space for canonical seed matches than the ∼21 kb ORF1ab region, so the absence of high-confidence 3′UTR sites among the validated miRNA set partly reflects sequence-space constraints. Second, the region-stratified thermodynamic analysis (Section 3.2.1) indicates that the SARS-CoV-2 3′UTR and CDS have similar mean MFE distributions (−10.40 *versus* −9.69 kcal/mol), which argues against any intrinsic thermodynamic preference for CDS targeting in this virus. Third, for endogenous mammalian mRNAs, 3′UTR-bound miRNAs generally exert stronger translational repression than CDS-bound miRNAs, owing to reduced interference from translating ribosomes at 3′ sites (Grimson et al., 2007; Gu et al., 2009; Hausser et al., 2013); however, for +ssRNA viruses this asymmetry is less firmly established, because viral genomes serve simultaneously as translation templates and as substrates for replication and packaging, and functional miRNA-mediated suppression through CDS binding has been documented across multiple +ssRNA virus families (Trobaugh and Klimstra, 2017; Bernier and Sagan, 2018; Girardi et al., 2018). Taken together, these considerations indicate that the observed CDS distribution reflects a combination of sequence-space constraints and the specific composition of the validated miRNA set, rather than a generalisable mechanistic preference for CDS targeting by antiviral miRNAs.

**FIGURE 9:**
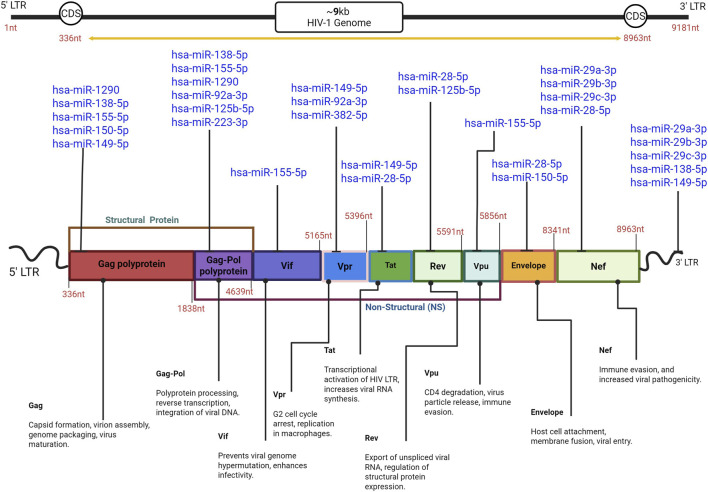
Predicted miRNA binding site distribution across HIV-1 coding regions.

For HIV-1, the experimentally validated inhibitory miRNAs demonstrated more complex targeting patterns reflecting the compact, overlapping viral genome organization (Figure 9). hsa-miR-149-5p exhibited the strongest predicted binding energy among the validated set (MFE = −17.0 kcal/mol), with high-confidence binding sites in the Tat and Vpr coding regions as well as the 3′UTR. hsa-miR-28-5p demonstrated the broadest targeting capability, with high-confidence binding sites spanning four distinct viral regions (Envelope, Nef, Rev, and Tat), including the strongest Nef interaction in the validated set (MFE = −16.0 kcal/mol). hsa-miR-138-5p exhibited high-confidence targeting across three regions (3′UTR, Gag-Pol, and Gag), while hsa-miR-125b-5p targeted both the Gag-Pol polyprotein and Rev. All three members of the experimentally validated miR-29 family, hsa-miR-29a-3p, hsa-miR-29b-3p, and hsa-miR-29c-3p, demonstrated identical dual-targeting patterns with binding sites in both the 3′UTR and Nef protein coding sequence, with MFE values of −13.1 kcal/mol for the 3′UTR site and −13.4 kcal/mol for the Nef site. Target region distribution among the 19 high-confidence sites revealed that the 3′UTR accounted for the largest number of high-confidence interactions (5), followed by Nef (4), Gag-Pol (3), Tat and Rev (2 each), and Envelope, Gag, and Vpr (1 each). The prominence of the 3′UTR among high-confidence targets despite its relatively small genomic fraction underscores the regulatory importance of this region in HIV-1 replication control.

### 3.6 Prediction of dual-targeting miRNAs with high-confidence binding energy

Within the high-confidence binding landscape defined above, a subset of experimentally validated antiviral miRNAs exhibited dual- or multi-targeting behavior, with predicted binding sites distributed across multiple viral genomic regions. These miRNAs demonstrated the capacity to simultaneously engage distinct viral genes through energetically favorable interactions, suggesting potential for coordinated antiviral regulation.

For SARS-CoV-2, several validated miRNAs displayed high-confidence multi-region targeting patterns. hsa-miR-15a-5p and hsa-miR-29a-3p each showed the most extensive profiles, with high-confidence binding sites spanning four viral regions: hsa-miR-15a-5p targeted the polyprotein, Spike, Nucleoprotein, and ORF3a, while hsa-miR-29a-3p targeted the polyprotein, Spike, Nucleoprotein, and Membrane protein. hsa-miR-497-5p demonstrated high-confidence interactions across three regions (polyprotein, Nucleoprotein, and ORF3a). Four additional miRNAs exhibited dual-region high-confidence targeting: hsa-miR-145-5p (polyprotein and Membrane), hsa-miR-298 (polyprotein and Spike), hsa-miR-24-3p (polyprotein and Spike), and hsa-miR-138-5p (polyprotein and Spike). These observations indicate that a substantial subset of host miRNAs can engage multiple essential SARS-CoV-2 gene products through stable binding interactions.

In contrast, HIV-1 exhibited an even broader spectrum of high-confidence multi-region targeting miRNAs, consistent with its compact and overlapping genome organization. hsa-miR-28-5p demonstrated the most extensive targeting profile, with high-confidence binding sites spanning four regions (Envelope, Nef, Rev, and Tat). Two miRNAs exhibited targeting across three regions: hsa-miR-138-5p (3′UTR, Gag-Pol, and Gag) and hsa-miR-149-5p (3′UTR, Tat, and Vpr). All three members of the miR-29 family (hsa-miR-29a-3p, hsa-miR-29b-3p, and hsa-miR-29c-3p) consistently exhibited dual-targeting of the 3′UTR and Nef, reinforcing their established role in HIV-1 restriction. Additionally, hsa-miR-125b-5p targeted both the Gag-Pol polyprotein and Rev.

Quantitatively, all seven HIV-1 miRNAs with high-confidence predictions exhibited multi-region targeting (100%), whereas SARS-CoV-2 supported multi-region targeting for seven of twelve miRNAs with high-confidence predictions (58.3%). This striking difference in multi-region targeting prevalence reflects the compact and overlapping organisation of the HIV-1 genome relative to SARS-CoV-2, which provides greater opportunity for a single miRNA seed to engage binding sites across multiple overlapping reading frames. The complete multi-region targeting observed for HIV-1 high-confidence miRNAs further suggests that the most thermodynamically stable antiviral miRNA interactions preferentially target multiple viral gene products, a property that may contribute to the potency of miRNA-mediated viral restriction.

Together, these high-confidence dual-targeting patterns highlight virus-specific differences in miRNA targeting architecture, with HIV-1 displaying a greater prevalence of energetically stable multi-region interactions compared to SARS-CoV-2.

### 3.7 Horizontal benchmarking against miRanda and RNAhybrid

To evaluate the predictive performance of miRNAProtPred relative to established sequence-based miRNA target prediction tools, a focused validation-based benchmark was conducted using miRanda v3.3a and RNAhybrid v2.1.2 as described in Section 2.8. The same sets of experimentally validated antiviral miRNAs (16 for SARS-CoV-2 and 13 for HIV-1) were submitted to all three tools against identical target sequences comprising the 3′UTR regions and complete coding sequences of both viruses. miRanda was executed using a score threshold ≥140 and energy threshold ≤−12.0 kcal/mol, restricting predictions to thermodynamically stable interactions comparable to the high-confidence tier of miRNAProtPred. RNAhybrid was executed using the 3utr_human background distribution and evaluated both under the conventional significance threshold (p ≤ 0.05) and without statistical filtering (raw predictions). Unless otherwise stated, comparative MFE analyses were based on the strongest (lowest-energy) interaction recovered for each validated miRNA.

For SARS-CoV-2, miRNAProtPred in strict mode recovered all 16 validated antiviral miRNAs (100% recall), identifying 97 predicted binding sites across the 3′UTR and coding sequence regions. miRanda recovered 15 of the 16 validated miRNAs (93.8%), generating 69 binding sites, including 68 within coding regions and one within the 3′UTR. The only miRNA not recovered by miRanda was hsa-miR-223-3p, which miRNAProtPred identified with three predicted binding sites in the polyprotein coding region (best MFE = −10.0 kcal/mol; Medium confidence). This interaction fell below the miRanda alignment score threshold, consistent with its comparatively weaker thermodynamic stability. Among the 15 miRNAs recovered by both tools, miRanda generated an average of 4.6 binding sites per miRNA, with the largest candidate sets observed for hsa-miR-15a-5p (14 sites), hsa-miR-497-5p (13 sites), and hsa-miR-29a-3p (10 sites). The mean best-site MFE across the 15 SARS-CoV-2 miRNAs recovered by both tools was −21.20 kcal/mol for miRanda, compared with approximately −12.0 kcal/mol for miRNAProtPred. This difference reflects both miRanda’s thermodynamic filtering criteria and its alignment-based scoring strategy, which extends beyond the seed-matched region to include supplementary 3′ compensatory pairing, thereby producing systematically more negative MFE values.

RNAhybrid without statistical filtering returned one best-fit hybridization for all 16 validated SARS-CoV-2 miRNAs in both the 3′UTR and CDS regions (32 predictions total). However, application of the conventional significance threshold (p ≤ 0.05) reduced recovery to only three miRNAs (18.8%): hsa-miR-1909-3p, hsa-miR-128-1-5p, and hsa-miR-365b-5p, all detected in the 3′UTR. No CDS interactions achieved statistical significance. Relaxation of the threshold to p ≤ 0.10 recovered one additional miRNA (hsa-miR-298; p = 0.071), while p ≤ 0.20 recovered one further candidate (hsa-miR-138-5p; p = 0.144). The uniformly non-significant CDS p-values likely reflect a known limitation of RNAhybrid’s statistical framework, which was calibrated using human 3′UTR background distributions. When applied to long viral coding sequences, the null model becomes increasingly conservative because longer targets provide greater opportunity for random low-energy hybridizations, thereby suppressing statistical significance despite thermodynamically favorable duplex formation (Kruger and Rehmsmeier, 2006). Consequently, RNAhybrid MFE values remain informative for thermodynamic ranking, but CDS-associated p-values should be interpreted cautiously in viral genome analyses.

For HIV-1, miRNAProtPred achieved 84.6% recall in strict mode (11/13) and 100% recall when strict and relaxed modes were combined (13/13), yielding 34 and 37 predicted binding sites, respectively. The two miRNAs requiring relaxed-mode recovery, hsa-miR-92a-3p and hsa-miR-382-5p, are discussed in Section 3.4. miRanda recovered 10 of the 13 validated HIV-1 miRNAs (76.9%), generating 16 binding sites, including five in the 3′UTR and 11 in coding regions. Three experimentally validated miRNAs were not recovered by miRanda: hsa-miR-125b-5p, hsa-miR-92a-3p, and hsa-miR-382-5p. Notably, hsa-miR-125b-5p was identified by miRNAProtPred in strict mode as a high-confidence candidate (best MFE = −14.1 kcal/mol) with predicted binding sites in the Gag-Pol and Rev coding regions. Huang et al. (2007) demonstrated that hsa-miR-125b-5p contributes to HIV-1 latency maintenance in resting CD4^+^ T cells, suggesting that canonical seed complementarity at this locus is sufficient for biologically relevant targeting despite the absence of extended alignment support required by miRanda. In contrast, miRanda recovered hsa-miR-223-3p and hsa-miR-150-5p with strong alignment scores (154 and 147, respectively) and MFE values of −16.13 and −17.68 kcal/mol, consistent with their classification as medium-confidence miRNAProtPred candidates.

Among HIV-1 3′UTR interactions, the hsa-miR-29 family members exhibited the strongest miRanda-predicted binding energies (hsa-miR-29a-3p: −25.57 kcal/mol; hsa-miR-29b-3p: −26.59 kcal/mol; hsa-miR-29c-3p: −22.87 kcal/mol), independently supporting both their high-confidence classification by miRNAProtPred and their experimentally established roles as HIV-1 restriction factors (Nathans et al., 2009). The mean best-site MFE across the 10 HIV-1 miRNAs recovered by miRanda was −20.59 kcal/mol, compared with −12.13 kcal/mol for miRNAProtPred. As observed for SARS-CoV-2, this difference primarily reflects miRanda’s broader alignment-based energy calculation. RNAhybrid without statistical filtering returned predictions for all 13 validated HIV-1 miRNAs (26 predictions total). Under the p ≤ 0.05 threshold, three miRNAs achieved significance in the 3′UTR: hsa-miR-29a-3p (p = 0.019), hsa-miR-29b-3p (p = 0.012), and hsa-miR-138-5p (p = 0.046), corresponding to a recovery rate of 23.1% (3/13). Several additional miRNAs approached significance, including hsa-miR-149-5p (p = 0.052), hsa-miR-29c-3p (p = 0.063), hsa-miR-382-5p (p = 0.069), and hsa-miR-92a-3p (p = 0.078). Relaxation of the threshold to p ≤ 0.10 increased 3′UTR recovery to 8/13 (61.5%), suggesting that the HIV-1 3′UTR contains a greater density of statistically supported miRNA interaction sites relative to the SARS-CoV-2 3′UTR under the human background model. As with SARS-CoV-2, no HIV-1 CDS predictions achieved statistical significance.

Across both viral systems, miRNAProtPred recovered 27 of 29 experimentally validated antiviral miRNAs in strict mode (93.1%) and all 29 in combined strict and relaxed analysis (100%). miRanda recovered 25 of 29 validated miRNAs (86.2%), whereas RNAhybrid achieved complete recovery only when statistical filtering was removed; under the conventional p ≤ 0.05 threshold, RNAhybrid recovered only 6 of 29 validated miRNAs (20.7%). These findings demonstrate that miRNAProtPred achieves the highest validated miRNA recovery while maintaining biologically interpretable seed-based targeting constraints. The four validated miRNAs recovered by miRNAProtPred but not by miRanda (hsa-miR-223-3p in SARS-CoV-2; hsa-miR-125b-5p, hsa-miR-92a-3p, and hsa-miR-382-5p in HIV-1) highlight two important mechanistic differences between the tools: first, canonical seed-level complementarity can support biologically relevant targeting even without extended alignment support; second, wobble-permissive relaxed matching can recover experimentally validated interactions missed by stricter alignment-based approaches. No validated miRNA was recovered exclusively by miRanda, indicating that the validated interactions accessible to miRanda’s alignment framework were fully recoverable through miRNAProtPred combined strict and relaxed analysis.

When candidate list sizes were compared at matched thermodynamic thresholds, miRanda generated 69 SARS-CoV-2 binding sites with MFE ≤ −12.0 kcal/mol, compared with 46 high-confidence miRNAProtPred sites within the same energy range, corresponding to a 1.5-fold expansion of the candidate list. This increase reflects miRanda’s allowance of G:U wobble pairing, mismatches, and gap formation within extended alignments. For HIV-1, miRanda generated 16 binding sites compared with 19 high-confidence miRNAProtPred sites, corresponding to a relative reduction in candidate number (0.84-fold). This difference was driven primarily by the failure of miRanda to recover several experimentally validated interactions detected by miRNAProtPred, particularly hsa-miR-125b-5p. These observations indicate that the relationship between matching permissiveness and candidate expansion is context-dependent and may vary according to the genomic architecture of the viral target.

An important methodological distinction should be considered when interpreting MFE values across the three tools. miRNAProtPred computes MFE using ViennaRNA RNAduplex for a defined complementary target site anchored at the seed match, producing an energy estimate specific to the predicted duplex region. miRanda computes MFE across an extended gapped alignment that may include compensatory 3′ pairing, resulting in systematically lower energies. RNAhybrid identifies the global minimum free-energy duplex within the full target sequence without requiring canonical seed complementarity, typically yielding the most negative energies among the three approaches. Consequently, direct numerical comparison of MFE values across tools is not biologically meaningful; instead, MFE values should be interpreted within the context of each tool’s scoring framework and used primarily for within-tool ranking of candidate interactions. A feature-level comparison summarizing workflow and prediction characteristics across the three tools is presented in Table 1. Complete benchmarking results, including binding site coordinates, alignment scores, MFE values, and RNAhybrid p-values, are provided in Supplementary Material S5.

### 3.8 Large-scale performance evaluation on the miRAW benchmark dataset

To provide a comprehensive assessment of classification performance beyond virus-specific validation, miRNAProtPred was evaluated against the miRAW benchmark dataset, a large-scale collection of experimentally validated human miRNA-mRNA interactions comprising 62,215 miRNA-target pairs with balanced class distribution (32,075 positive interactions, 51.56%; 30,140 negative interactions, 48.44%). This evaluation provides an independent, unbiased assessment of predictive performance on endogenous human miRNA-target interactions that were not used in the development or parameterization of miRNAProtPred.

In strict mode, miRNAProtPred achieved a precision of 98.66% (23,727 true positives out of 24,050 positive predictions), indicating that positive predictions are almost exclusively correct. Recall was 73.97% (23,727 of 32,075 actual positive interactions recovered), reflecting the conservative design of the canonical seed-matching criterion. Specificity was 98.93% (29,817 of 30,140 negative interactions correctly classified), confirming that the tool generates very few false positive predictions (323 across the entire dataset). Overall accuracy was 86.06%, the F1-score was 0.846, the Matthews correlation coefficient (MCC) was 0.748, and the area under the receiver operating characteristic curve (ROC-AUC) was 0.865. To quantify the sensitivity gain associated with wobble-permissive matching, the same evaluation was repeated in relaxed mode. Relaxed mode increased recall from 73.97% to 75.53%, recovering an additional 499 true positive interactions (24,226 total), while precision decreased from 98.66% to 96.58%, with 535 additional false positives (858 total). The overall F1-score remained essentially unchanged (0.848 vs. 0.846), as did accuracy (86.01% vs. 86.06%) and MCC (0.740 vs. 0.748). The near-equivalence of the number of additional true positives (499) and additional false positives (535) indicates that wobble-permissive matching on this dataset operates near the break-even point of the precision-recall trade-off, where each additional correct recovery is accompanied by approximately one additional incorrect prediction. This quantitative result supports the default use of strict mode for applications where prediction reliability is prioritized, while relaxed mode provides a modest sensitivity extension at the cost of reduced precision.

These metrics are consistent with the precision-oriented design philosophy of miRNAProtPred. The high precision and specificity in strict mode confirm that canonical seed matching with thermodynamic evaluation produces a highly reliable positive prediction set, whereas the moderate recall reflects the deliberate exclusion of non-canonical interactions from the default search. The 26% of positive interactions not recovered in strict mode are expected to include targets mediated through non-canonical binding, cooperative miRNA activity, or interactions dependent on supplementary 3′ pairing that is not captured by the seed-centric prediction framework. The MCC of 0.748, which accounts for all four confusion matrix categories and is robust to class imbalance, provides a single summary metric indicating strong overall classification quality. Complete classification metrics for both modes are summarized in Table 3.

**TABLE 3:** Classification performance of miRNAProtPred on the miRAW benchmark dataset.

Metric	Strict mode	Relaxed mode	Difference
True Positives	23,727	24,226	+499
True Negatives	29,817	29,282	−535
False Positives	323	858	+535
False Negatives	8,348	7,849	−499
Precision	98.66%	96.58%	−2.08 pp
Recall	73.97%	75.53%	+1.56 pp
Specificity	98.93%	97.15%	−1.78 pp
Accuracy	86.06%	86.01%	−0.05 pp
F1-score	0.846	0.848	+0.002
MCC	0.748	0.740	−0.008
ROC-AUC	0.865	0.863	−0.002

## 4 Discussion

Beyond predictive performance, miRNAProtPred addresses a practical workflow-integration gap that has received limited attention in the miRNA target prediction literature. Whereas established tools such as miRanda and RNAhybrid provide robust thermodynamic and sequence-based prediction engines, their deployment requires users to independently compile miRNA sequence files, format target inputs, execute predictions on a per-miRNA basis or through custom scripting, and apply *post hoc* filtering thresholds, a multi-step process that introduces variability across laboratories and limits reproducibility (Riffo-Campos et al., 2016). By consolidating these steps into a single command-line interface with a bundled database of 2,656 curated human miRNAs, automatic sequence type detection, protein-to-nucleotide conversion *via* tblastn, and structured output with multi-criteria confidence classification, miRNAProtPred reduces the practical barrier to entry for researchers investigating non-canonical targets such as viral genomes, cross-species interactions, and protein-derived sequences. The automated integration of miRNA sequences from miRDB and miRBase eliminates the need for users to obtain, format, or maintain external FASTA files, while the protein-input pathway provides a capability absent from all currently available prediction tools, enabling researchers working with protein-centric annotation databases such as UniProt to perform miRNA target prediction without manual nucleotide sequence retrieval. This design philosophy prioritizes standardization and accessibility rather than algorithmic novelty, and the tool is intended to complement rather than replace more specialized prediction platforms.

Validation against experimentally confirmed antiviral miRNAs from two evolutionarily distinct viral families demonstrated that the dual-mode architecture achieves comprehensive sensitivity while preserving the precision advantages of the default strict mode. Strict canonical matching alone achieved complete recovery for SARS-CoV-2 and recovered the large majority of validated HIV-1 miRNAs, while the wobble-permissive relaxed mode recovered the remaining two HIV-1 miRNAs that were inaccessible through canonical seed complementarity. The recovery of hsa-miR-382-5p with marginal binding energy through relaxed mode is particularly informative, as its experimentally demonstrated contribution to HIV-1 latency is mediated through cooperative activity with four other miRNAs enriched in resting CD4^+^ T cells (Huang et al., 2007) rather than through independent high-affinity canonical targeting. The appropriately low confidence classification assigned to this interaction by the multi-criteria framework illustrates that the tool can detect cooperatively acting miRNAs while correctly flagging them as low-confidence individual interactions, preserving the discriminatory value of the tiered scoring system.

The *a priori* −12 kcal/mol threshold for high-confidence classification (Hsu, 2006; Hsu et al., 2011) was independently supported by the thermodynamic enrichment observed among validated miRNAs relative to genome-wide prediction backgrounds in both viral systems. Importantly, this threshold retains discriminatory power across viral families with distinct nucleotide compositions, classifying 75% and 63.6% of validated miRNAs as high confidence for SARS-CoV-2 and HIV-1, respectively, despite the narrower enrichment window in the A/U-rich HIV-1 genome. The smaller enrichment magnitude for HIV-1 reflects its higher baseline thermodynamic stability, likely attributable to greater uridine content favouring A-U-rich duplex formation and a longer evolutionary co-existence with the human host that has shaped the density of accessible single-stranded regions in its genome.

Comparative analysis revealed that miRNA targeting architecture is shaped by virus-specific genomic organisation rather than universal targeting rules: SARS-CoV-2 prediction counts are driven predominantly by sequence-length effects, whereas the HIV-1 3′UTR exhibits genuine enrichment for thermodynamically stable binding beyond what length alone would predict (Nathans et al., 2009; Houzet and Jeang, 2011). The complete multi-region targeting observed for all seven high-confidence HIV-1 validated miRNAs, compared with 58.3% for SARS-CoV-2, further suggests that the most potent antiviral miRNAs preferentially engage multiple viral gene products, a property with direct implications for therapeutic candidate prioritisation.

The precision-recall trade-off inherent in seed-matching stringency was quantified through large-scale evaluation on the miRAW benchmark dataset (62,215 experimentally validated miRNA-target pairs). Strict mode achieved 98.66% precision and 73.97% recall (F1 = 0.846; MCC = 0.748), confirming that canonical seed matching with thermodynamic evaluation produces a highly reliable positive prediction set. Relaxed mode modestly increased recall to 75.53% at the cost of a precision reduction to 96.58%, with the near-equivalence of additional true positives (499) and additional false positives (535) indicating that wobble-permissive matching operates near the break-even point of the precision-recall trade-off on endogenous datasets. The MCC, which accounts for all four confusion matrix categories and is robust to class imbalance, provides a single summary metric indicating strong overall classification quality. The 26% of positive interactions not recovered in strict mode are expected to include targets mediated through non-canonical binding, cooperative miRNA activity, or supplementary 3′ pairing not captured by the seed-centric prediction framework. These results support the default use of strict mode for applications where prediction reliability is prioritised, while relaxed mode provides a biologically informed sensitivity extension for comprehensive screening or for targets where non-canonical miRNA-mediated regulation is suspected.

Benchmarking against miRanda and RNAhybrid demonstrated that miRNAProtPred achieves superior recovery of validated antiviral miRNAs compared to both established tools. For SARS-CoV-2, miRNAProtPred in strict mode recovered all 16 validated miRNAs (100%), exceeding miRanda (93.8%) and statistically filtered RNAhybrid (18.8%). For HIV-1, combined strict and relaxed analysis achieved complete recovery (100%), compared with miRanda (76.9%) and RNAhybrid at p ≤ 0.05 (23.1%). Four validated miRNAs were recovered exclusively by miRNAProtPred and not by miRanda (hsa-miR-223-3p in SARS-CoV-2; hsa-miR-125b-5p, hsa-miR-92a-3p, and hsa-miR-382-5p in HIV-1), whereas no validated miRNA was recovered exclusively by miRanda, indicating that the validated interactions accessible to miRanda’s alignment framework were fully recoverable through miRNAProtPred’s combined analysis. At matched thermodynamic thresholds, miRanda generated 1.5-fold more SARS-CoV-2 candidate binding sites than miRNAProtPred, reflecting its allowance of G:U wobble pairing, mismatches, and gap formation within extended alignments. RNAhybrid’s conservative statistical model, calibrated on human transcriptome characteristics, was the least effective on viral targets, with CDS p-values uniformly failing to reach significance due to length-dependent saturation of the background distribution (Kruger and Rehmsmeier, 2006). These findings highlight the limitations of human-trained statistical frameworks when applied to non-canonical genomic contexts.

Several limitations should be acknowledged. First, thermodynamic scoring *via* RNAduplex does not account for local target-site accessibility, meaning predictions within highly structured genomic regions, such as viral 5′UTRs, may be thermodynamically favourable in isolation but structurally inaccessible *in vivo*. Users should exercise caution when interpreting predictions in extensively folded regulatory regions such as viral 5′UTRs. Second, the current framework does not incorporate evolutionary conservation, cooperative binding effects, or the well-established 3′UTR positional bias in endogenous mammalian contexts (Grimson et al., 2007); users interpreting predictions in endogenous gene regulation settings should apply these priors accordingly. Third, the protein-input pathway is best regarded as a convenience feature; users requiring reference-consistent predictions should supply a verified nucleotide sequence directly, particularly for proteins encoded by spliced mRNAs. Fourth, validation was restricted to two viral pathogens, and extension to additional biological contexts will further establish generalisability. Fifth, the multi-criteria confidence classification thresholds (seed length, AU context, motif identity) were informed by published threshold ranges in the miRNA targeting literature (Lewis et al., 2005; Grimson et al., 2007; Agarwal et al., 2015); rather than derived from a training set; users may adjust the analytical stringency through the output mode system (highconf, concise, raw) to accommodate different analytical objectives. We also acknowledge that a subset of the miRNAs in our viral reference validation set may have been originally prioritised through seed-complementarity or thermodynamic predictions, which could inflate the apparent sensitivity of miRNAProtPred relative to an entirely independently validated benchmark. The recovery rates reported from the viral validation should therefore be interpreted as sensitivity estimates rather than unbiased performance metrics. This potential circularity concern is mitigated by the independent evaluation on the miRAW benchmark dataset, which comprises experimentally validated interactions not selected through seed-based computational approaches, and on which miRNAProtPred achieved comparable performance metrics (precision 98.66%, MCC 0.748).

miRNAProtPred provides a standardised and accessible platform for prioritising human miRNA-target interactions across diverse genomic contexts. Its applicability to real biological problems is further supported by its use in related studies, including a recent computational analysis of Zika virus infection, where the tool was integrated into a multi-layered pipeline to identify dual-targeting miRNAs capable of simultaneously modulating viral transcripts and host proviral pathways (Dutta et al., 2026). The current version of miRNAProtPred incorporates a user-selectable seed-matching stringency parameter (--mode strict or--mode relaxed), enabling users to calibrate the precision-sensitivity balance according to their specific experimental throughput and biological context.

Several directions for future development are envisioned. First, the integration of target-site accessibility metrics derived from RNA secondary structure prediction (e.g., RNAplfold or RNAfold) would complement the current thermodynamic scoring with structural context, enabling the tool to distinguish between thermodynamically favourable binding sites that are structurally accessible and those that are occluded by local RNA folding, a consideration that is particularly relevant for highly structured viral 5′UTRs and long non-coding RNAs. Second, incorporating evolutionary conservation analysis across related viral strains or host species would provide an additional layer of functional evidence for predicted interactions. For viral targets, conservation of predicted binding sites across circulating strains could identify interactions that are robust to viral evolution and therefore more likely to represent durable therapeutic targets, whereas strain-specific binding-site losses could flag resistance-prone candidates. For endogenous targets, cross-species conservation of miRNA binding sites remains one of the strongest predictors of functional repression (Friedman et al., 2009), and its integration would improve the specificity of predictions in mammalian transcriptome contexts. Third, the current single-site prediction framework does not model cooperative or context-dependent binding effects, in which multiple miRNAs act synergistically on the same transcript or a single miRNA achieves functional repression only in the context of specific cellular conditions. The recovery of hsa-miR-382-5p with marginal binding energy (MFE = −6.7 kcal/mol) in the present study illustrates this limitation: its experimentally demonstrated contribution to HIV-1 latency is mediated through cooperative activity with four other miRNAs in resting CD4^+^ T cells (Huang et al., 2007), a mechanism that cannot be captured by independent single-site thermodynamic evaluation. Future versions could implement cooperative scoring models that assess combinatorial miRNA targeting density on individual transcripts, drawing on emerging frameworks for multi-miRNA target regulation (Sætrom et al., 2007; Rinck et al., 2013). Fourth, exploring a supervised learning layer that combines the seed-based and thermodynamic features currently computed by miRNAProtPred with contextual features such as target-site accessibility, flanking sequence composition, and binding-site clustering would enable the tool to leverage the growing body of experimentally validated cross-species and viral miRNA interaction data. Recent advances in deep learning architectures, including convolutional neural networks, transformer-based attention models, and graph neural networks, have demonstrated substantial improvements in endogenous miRNA target prediction by capturing complex sequence patterns and long-range dependencies that rule-based approaches cannot model (Cheng et al., 2016; Pla et al., 2018; Wen et al., 2018). While the current scarcity of validated non-canonical training data motivated the rule-based approach adopted in this work, databases such as miRTarBase continue to expand (Cui et al., 2025), and hybrid architectures that use seed-based features as structured inputs to deep learning models have demonstrated improved performance in endogenous target prediction contexts. As these datasets grow to encompass viral and cross-species interactions, a data-driven refinement of the confidence classification framework may further improve the balance between precision and sensitivity.

## 5 Conclusion

miRNAProtPred demonstrates that canonical seed matching complemented by thermodynamic ranking and multi-criteria confidence classification can be delivered as a self-contained, locally executable package that eliminates the manual preprocessing burden associated with existing miRNA target prediction workflows. The user-selectable dual-mode architecture, combining strict canonical matching with wobble-permissive relaxed analysis, achieved complete recovery of experimentally validated antiviral miRNAs across both SARS-CoV-2 and HIV-1, while large-scale evaluation on the miRAW benchmark dataset confirmed strong classification performance with high precision and robust MCC. Comparative benchmarking established that miRNAProtPred recovers validated miRNAs missed by both miRanda and RNAhybrid, with no validated interaction recoverable exclusively by either competing tool. The cross-virus comparison further demonstrates that miRNA-binding landscapes are shaped by virus-specific genomic architecture rather than universal targeting rules, arguing against transferring candidate-selection strategies across viral families without empirical evaluation. Together, these findings support the use of miRNAProtPred as a first-pass screening and prioritisation tool for host-pathogen miRNA interactions, RNA-based therapeutics, and antiviral miRNA discovery, while recognising that predictions require experimental validation and should be complemented by structural accessibility analysis for targets with extensively folded regulatory regions.

## Data Availability

The original contributions presented in the study are included in the article/Supplementary Material, further inquiries can be directed to the corresponding authors.
